# Comprehensive insight into anti-staphylococcal and anti-enterococcal action of brominated and chlorinated pyrazine-based chalcones

**DOI:** 10.3389/fmicb.2022.912467

**Published:** 2022-08-17

**Authors:** Klára Konečná, Adéla Diepoltová, Pavlína Holmanová, Ondřej Jand’ourek, Marcela Vejsová, Barbora Voxová, Pavel Bárta, Jana Maixnerová, František Trejtnar, Marta Kučerová-Chlupáčová

**Affiliations:** ^1^Department of Biological and Medical Sciences, Faculty of Pharmacy in Hradec Králové, Charles University, Hradec Králové, Czechia; ^2^Department of Biophysics and Physical Chemistry, Faculty of Pharmacy in Hradec Králové, Charles University, Hradec Králové, Czechia; ^3^Department of Pharmacology and Toxicology, Faculty of Pharmacy in Hradec Králové, Charles University, Hradec Králové, Czechia; ^4^Department of Pharmaceutical Chemistry and Pharmaceutical Analysis, Faculty of Pharmacy in Hradec Králové, Charles University, Hradec Králové, Czechia

**Keywords:** halogenated chalcones, pyrazine-based chalcones, methicillin- and vancomycin-resistant *Staphylococcus aureus*, coagulase-negative staphylococci, multidrug-resistant enterococci, checkerboard assays, *in vivo* toxicity, post-antimicrobial effect

## Abstract

The greatest threat and medicinal impact within gram-positive pathogens are posed by two bacterial genera, *Staphylococcus* and *Enterococcus*. Chalcones have a wide range of biological activities and are recognized as effective templates in medicinal chemistry. This study provides comprehensive insight into the anti-staphylococcal and anti-enterococcal activities of two recently published brominated and chlorinated pyrazine-based chalcones, CH-0y and CH-0w. Their effects against 4 reference and 12 staphylococcal and enterococcal clinical isolates were evaluated. Bactericidal action, the activity in combination with selected conventional antibiotics, the study of post-antimicrobial effect (PAE, PAE/SME), and *in vitro* and *in vivo* toxicity, were included. In CH-0y, anti-staphylococcal activity ranging from MIC = 15.625 to 62.5 μM, and activity against *E. faecium* from 31.25 to 62.5 μM was determined. In CH-0w, anti-staphylococcal activity ranging from 31.25 to 125 μM, and activity against *E. faecium* and *E. faecalis* (62.5 μM) was revealed. Both CH-0y and CH-0w showed bactericidal action, beneficial impact on bacterial growth delay within PAE and PAE/SME studies, and non/low toxicity *in vivo*. Compared to CH-0w, CH-0y seems to have higher anti-staphylococcal and less toxic potential. In conclusion, chalcones CH-0y and CH-0w could be considered as structural pattern for future adjuvants to selected antibiotic drugs.

## Graphical Abstract

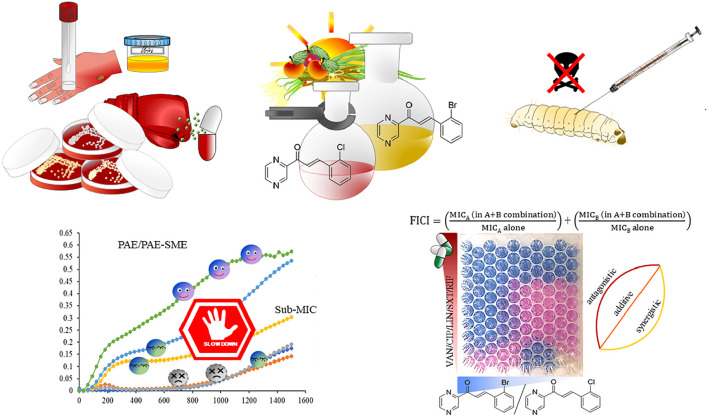

## Introduction

Without any objection, antibiotic resistance is considered as one of the most significant challenges to modern medicine. The phenomenon of antibiotic resistance has significant consequences, in which especially factors such as complicated treatment, higher health costs, and higher mortality have the largest share (Barsoumian et al., 2015; Friedman et al., 2016). The situation has aggravated so much that it is described as entirely critical. Some factors have indirectly contributed to this crisis, including among others, a lack of effective antimicrobials in clinical trials and a lack of anti-infective drug development in the pharmaceutical industry (World Health Organization [WHO], 2021). Therefore, one of the options to manage this stalemate is to place greater emphasis on the discovery and research of new anti-infectives, and speed up the search for alternative approaches to combat infectious agents.

The greatest threat and medicinal impact among the gram-positive pathogens are posed by two bacterial genera, *Staphylococcus* and *Enterococcus* (Ventola, 2015). Within the genus *Staphylococcus*, the most frequently mentioned are methicillin-resistant *Staphylococcus aureus* (MRSA) and vancomycin-resistant *Staphylococcus aureus* (VRSA) strains (Chambers and Deleo, 2009; McGuinness et al., 2017). These strains have developed resistance to conventional antibiotics from different groups, e.g., fluoroquinolones, aminoglycosides, macrolides, etc. (Kaur and Chate, 2015). However, in the context of medical significance, other representatives of this genus, coagulase-negative staphylococci (CoNS), cannot be overlooked either. CoNS are recognized as the main nosocomial pathogens. CoNS infections are mostly less severe than *Staphylococcus aureus* (SA) infections (Heilmann et al., 2019). On the other hand, their treatment is usually more complicated because of the dramatic emergence of antibiotic resistance. In the antibiogram profile of these bacteria, the resistance against the action of oxacillin/methicillin, gentamicin, ciprofloxacin, clindamycin, and even linezolid has been reported (Becker et al., 2014; Moawad et al., 2019). The second mentioned bacterial genus, *Enterococcus*, represents a group of bacteria that show significant potential to acquire multidrug resistance determinants quickly (Ahmed and Baptiste, 2018). The collection of anti-infective drugs to which they may be resistant is extensive and includes the antimicrobials of last-resort (linezolid, daptomycin, tigecycline, etc.) acting against glycopeptide (vancomycin) and multidrug-resistant (MDR) bacterial strains, as well (Miller et al., 2014; Ahmed and Baptiste, 2018; Bender et al., 2018). Both staphylococci and enterococci belong to the MDR pathogens, also named superbugs. Along with other superbugs, such as *Klebsiella pneumoniae*, *Acinetobacter baumannii*, *Pseudomonas aeruginosa*, and *Enterobacter* species, they are included in the ESKAPE group of pathogens, which are the leading cause of nosocomial infections throughout the world (Khan and Khan, 2016). The emphasis on the clinical significance of these pathogens is demonstrated by the declaration of the World Health Organization (WHO). In the WHO declaration, staphylococci and enterococci are registered as “priority pathogens” that pose the greatest threat to human health. WHO demarcates the antibacterial research being conducted against these microbial entities as one of its priorities (World Health Organization [WHO], 2017).

Chalcones are recognized in medicinal chemistry as privileged structure compounds and effective templates in drug discovery. These compounds have a core chemical pattern of 1,3-diphenylprop-2-en-1-one and have attracted a great deal of interest, especially due to their multitargeted and broad spectrum of interesting biological activities (Zhuang et al., 2017; Xu et al., 2019). The list of these activities includes, for example, anticancer, anti-inflammatory, antibacterial, antiviral, antiparasitic, antioxidant, and others (Mahapatra et al., 2017; Xu et al., 2019; Elkhalifa et al., 2021; Ouyang et al., 2021).

Historically, within anti-infective drug discovery research, various antibiotic natural products have brought unfailing inspiration and have played a crucial role in the development of new antimicrobial compounds (Abouelhassan et al., 2019). With regard to antibacterial activities, xanthohumol and desmethylxanthohumol (Supplementary Figure 1), two naturally occurring and isolated from hop (*Humulus lupulus*, *Cannabaceae*), are among the best chalcones that have been studied in depth. The recognized activity of these compounds against MRSA, expressed by minimum inhibitory concentration (MIC), is in the range of 9.8–19.5 and 19.5–39 μg/mL, respectively (Bocquet et al., 2019). Another structurally related natural chalcone, isobavachalcone (Supplementary Figure 1) from *Dorstenia bacteri* (*Moraceae*), has exhibited activity against SA, corresponding to MIC = 0.3 μg/mL (Mbaveng et al., 2008). The licochalcone A and C (Supplementary Figure 1) isolated from *Glycyrrhiza inflata* (*Fabaceae*) have been seen to inhibit the growth of *S. aureus* at an MIC of 1.56 and 6.25 μg/mL, respectively (Haraguchi et al., 1998). The antibacterial activity of licochalcone A against MRSA strains corresponding to MIC = 16 μg/mL (Hatano et al., 2000), MIC = 6.25 μg/mL (Fukai et al., 2002), and MIC = 3 μg/mL (Tsukiyama et al., 2002) has been revealed in other studies, as well. Among other natural compounds, 4-hydroxyderricin (Supplementary Figure 1) isolated from *Angelica keiskei* (*Apiaceae*) should not be omitted in this short overview. It demonstrated the activity corresponding to an MIC below 5 μM (Battenberg et al., 2013).

Natural chalcones have shown potent anti-enterococcal activity, as well. Activity corresponding to MIC = 0.125–2 μg/mL was revealed in panduratin A, isolated from the rhizome of fingerroot (*Boesenbergia rotunda*) (Rukayadi et al., 2009), in chalcone and dihydrochalcone isolated from *Uvaria chamae* roots corresponding to MIC = 2.3 μg/mL (Koudokpon et al., 2018), for xanthoangelol isolated from fruits of *Amorpha fruticosa*, with a revealed activity within the range of MIC = 3.125–12.5 μM (Meier et al., 2019), and for licochalcone A with the anti-enterococcal activity of MIC = 6 μg/mL (Tsukiyama et al., 2002).

In this study, our attention was focused on two halogenated pyrazine-based chalcones, recognized and highlighted as being promising for advanced research due to their antibacterial activities in our previous study (Kucerova-Chlupacova et al., 2016). Namely, (2*E*)-3-(2-bromophenyl)-1-(pyrazin-2-yl)prop-2-en-1-one and (2*E*)-3-(2-chlorophenyl)-1-(pyrazin-2-yl)prop-2-en-1-one were selected for this detailed and comprehensive characterization of their *in vitro* antibacterial effects.

## Materials and methods

### Chemistry

The compounds were synthesized as described previously (Kucerova-Chlupacova et al., 2016). Their identity and purity were checked using nuclear magnetic resonance spectra (NMR), mass spectra (MS), and elemental analysis (EA).

^1^H- and ^13^C-NMR spectra were recorded at ambient temperature using a Jeol JNM-ECZ600R (Jeol Ltd., Tokyo, Japan) operating at 600 MHz for ^1^H and 151 MHz for ^13^C. Chemical shifts were recorded as values in ppm and were indirectly referenced to tetramethylsilane (TMS) *via* the solvent signal (7.26 for 1H, 77.0 for ^13^C in CDCl_3_). Coupling constants J are given in Hz. EA was performed using a Vario MICRO cube Element Analyzer (Elementar Analysensysteme, Hanau, Germany), and the values are given as percentages. The APCI-MS spectra in both positive and negative modes were measured using a single quad mass spectrometer (Expression, Advion, Inc., United States). The samples for MS were introduced as solids using an ASAP probe (Advion, Inc., United States).

### Evaluation of the activity against gram-positive reference strains, and clinical isolates

The microdilution broth method was performed according to EUCAST (The European Committee on Antimicrobial Susceptibility Testing) instructions (European Committee for Antimicrobial Susceptibility Testing [EUCAST] of the European Society for Clinical Microbiology Infectious Diseases [ESCMID], 2003), with slight modifications. Reference bacterial strains were purchased from the Czech Collection of Microorganisms (CCM, Brno, Czech Republic). Namely, the reference strains, *Staphylococcus aureus* subsp. *aureus* CCM 4223 (ATCC 29213), *Staphylococcus aureus* subsp. *aureus* methicillin-resistant (MRSA) CCM 4750 (ATCC 43300), *Enterococcus faecalis* CCM 4224 (ATCC 29212), and VRSA CCM 1767 were included in the antibacterial activity screening. Clinical isolates from the genus *Staphylococcus* and *Enterococcus* were kindly provided by the Department of Clinical Microbiology, University Hospital, Hradec Králové, Czechia. All these strains were taxonomically classified by biochemical tests and MALDI-TOF (Microflex LH/SH MALDI-TOF, Bruker Biotyper 3.0 SW, Bruker Daltonic GmbH, Bremen, Germany) instrumentation. The antibiograms of the strains were determined using the disc diffusion method in accordance with EUCAST recommendations (Matuschek et al., 2014). The clinical isolate, VRSA, was kindly provided by The National Institute of Public Health in Prague, Czech Republic. The list of reference strains and clinical isolates with susceptibility profiles and other strain specifications is shown in Supplementary Table 1.

Briefly, the cultivation was performed in Cation-adjusted Mueller-Hinton broth (CAMHB, M-H 2 Broth, Sigma-Aldrich, United States) at 35 ± 2°C. The tested compounds were dissolved in DMSO (Sigma-Aldrich, United States) to produce stock solutions. The final concentration of DMSO in the testing medium did not exceed 1% (v/v) of the total solution composition and did not affect the growth of bacteria. Both positive (microbe solely) and negative (cultivation medium and DMSO) controls and internal quality standards [ciprofloxacin, gentamicin, and vancomycin (Sigma-Aldrich, United States)] were involved in the assays. Antibacterial activity, expressed as MIC (reported in μM), was evaluated after 24 (48 h) of static incubation in a dark and humidified atmosphere at 35 ± 2°C. Visual inspection and spectrophotometric measurements (530 nm, Synergy HTX Multi-Mode Microplate reader, BioTek, United States) were used for making an MIC endpoint evaluation.

The concentration-dependent dynamics of the tested compound *vs.* selected staphylococcal or enterococcal strain were measured after 20-h incubation at 36.8^°^C by the instrument Bioscreen C (Oy Growth Curves, Finland, 540 nm).

### Checkerboard assays

A screening test for evaluating the antibacterial effect within mutual interaction (A + B) of two compounds with antimicrobial activity, tested compound (A), together with commercially available antibiotic drug (B), was performed using a broth microdilution checkerboard method. The assays were performed in Honeycomb plates (Oy Growth Curves, Finland) in a ten-by-ten well configuration. Five antibiotic agents used for the treatment of infections caused by gram-positive bacteria, namely, vancomycin (VAN), trimethoprim/sulfamethoxazole (SXT), rifampicin (RIF), ciprofloxacin (CIP), and linezolid (LIN) were selected for combinations (all purchased from Sigma-Aldrich, United States). Within the checkerboard assays, the MRSA ATCC 43300 strain was used for detecting the total antibacterial activity of the compounds in combination.

Briefly, the compounds were dissolved in DMSO (Sigma-Aldrich, United States) to produce stock solutions. The final concentration of DMSO in the testing medium did not exceed 1% (v/v) of the total solution composition and did not affect the growth of bacteria. The compounds were serially diluted (twofold) in CAMHB in separate microtiter wells and then transferred to a Honeycomb plate at a 1:1 volume ratio. The first compound was transferred in a horizontal direction, and the second one in a vertical direction. Each well with a final volume of 200 μl compound mixtures was inoculated with the MRSA bacterial strain (the final density corresponded to 5 × 10^6^ CFU/mL) in the CAMHB. The positive controls consisted solely of the test microbe, while the negative controls consisted of a cultivation medium, and compounds A and B alone were included in each assay, as well. The honeycomb plates were incubated in a Bioscreen C instrument (Oy Growth Curves, Finland) at 36.8^°^C for 20 h. At every 30 min of incubation, optical density (O.D.) was recorded at 580 nm. After incubation, the percentage inhibition in each well was compared to the positive controls (background values subtracted). A visual inspection and metabolic activity indicator, Alamar Blue (Alamar Blue TM Cell Viability reagent, Thermo Fisher Scientific, United States), were used for evaluating MIC endpoints.

The total fractional inhibitory concentration index (FICI) was used to interpret the checkerboard assays. The FICI was calculated according to the following formula: FICI = FIC(A) + FIC(B). FIC(A) and FIC(B) were calculated as follows: FIC (A or B) = MIC of drug A (or B) in combination/MIC of drug A (or B) alone. The results of the FICI were defined as follows: FICI ≤ 0.5, synergy; 0.5 < FICI ≤ 1, additivity; 1 < FICI ≤ 4, indifference; and FICI > 4, antagonism.

### Detection of the bacteriostatic vs. bactericidal mode of action

To distinguish between the bactericidal and bacteriostatic activity of CH-0y and CH-0w, three SA strains (131/16, 136/16, 137/16), the microdilution method, and subsequently the spread plate technique for colony forming units (CFU) evaluation were employed. Solutions of CH-0y and CH-0w in CAMHB with final concentrations ranging from 3.906 to 500 mM were prepared according to the procedure described above (section “Evaluation of the activity against gram-positive reference strains, and clinical isolates”). After incubation for 24 h, the MIC was evaluated. Further, the representative aliquots from wells where the concentrations of tested compounds corresponded to 4 × MIC were taken, serially diluted, seeded, and subcultured on Mueller-Hinton agar. Subsequently, agar plates were incubated for 24 h in a humid atmosphere, at 35 ± 2^°^C. Similarly, the initial bacterial inoculum was processed. After the incubation period, the number of CFU/mL was calculated, and minimum bactericidal concentration (MBC) was evaluated. MBC is defined as the lowest concentration of antimicrobial agent that leads to a reduction of initial bacterial inoculum viability corresponding to a value ≥ 99.9%. An antibacterial agent is usually regarded as bactericidal if the MBC is not higher than four times the MIC.

### Evaluation of the post-antimicrobial and post-antimicrobial sub-MIC effects of CH-0y and CH-0w, together with the involvement of the reference drugs, vancomycin, and ciprofloxacin

The MRSA strain ATCC 43300 was used in these experiments. The bacteria were grown to the early logarithmic phase in Mueller-Hinton broth at 37^°^C, with gentle shaking. Subsequently, bacterial suspension was diluted with fresh pre-heated CAMHB to a density corresponding to approximately 5 × 10^5^ CFU/mL and divided into aliquots in test tubes. The bacteria were subsequently harvested by a centrifugation step (10,000 × g, 12 min, 24^°^C), and the pellets were resuspended in test compounds/drug solutions in CAMHB at concentrations of 1 × MIC or 2 × MIC. The equivalent volumes of compounds/drugs solutions to the aliquots volumes in test tubes were used for resuspension. A drug-free control (positive growth control) was also included in the assay. Next, the bacteria were exposed to the tested compounds/drugs for 2 h, with cultivation at 37^°^C and gentle shaking. The exposed bacteria were then washed by centrifugation at 10,000 × g for 12 min and diluted in fresh CAMHB. The bacterial suspensions were transferred to Honeycomb plates in replicates. The method including the continuous mapping of the bacterial growth by a computerized incubator, Bioscreen C (540 nm), was employed for both post-antimicrobial (PAE) and post-antimicrobial sub-MIC (PAE-SME) evaluation (Löwdin et al., 1993).

For making a PAE-SME evaluation, the analogous procedure (cultivation of bacteria to the exponential phase, dilution of bacterial suspension, exposure to the tested compounds/drugs, harvesting and washing step, …), as mentioned above, was performed. After the harvesting and washing step, the equivalent volumes of compounds/drugs solutions (final concentrations of 0.8, 0.6, 0.4, 0.2 × MIC) to the aliquot’s volumes in test tubes before centrifugation were used for resuspension. Finally, the bacterial suspensions were transferred to Honeycomb plates in replicates, and the growth was continuously mapped by vertical photometry (540 nm) in a computerized incubator, Bioscreen C (Oy Growth Curves, Finland).

### Evaluation of CH-0y and CH-0w *in vitro* cytotoxicity

The human kidney epithelial cell line HK-2 purchased from American Type Culture Collection (ATCC, United States) was cultured in Dulbecco’s Modified Eagle’s Medium—high glucose (Sigma-Aldrich, United States) supplemented with 10% fetal bovine serum (Sigma-Aldrich, United States) and 1% L-glutamine solution (Sigma-Aldrich, United States) in a humidified atmosphere containing 5% CO_2_ at 37^°^C.

Briefly, the cells were seeded in density 15,000 cells per well in a 96-well plate 24 h prior to the experiment. The next day, the cells in triplicates were treated with the tested substances at a range of concentrations (1–250 μM). The controls representing 100% cell viability, 0% cell viability (the cells treated with 10% DMSO), no cell control, and vehiculum controls were incubated in parallel. After 24 h incubation in a humidified atmosphere containing 5% CO_2_ at 37^°^C, the reagent from the kit CellTiter 96 AQueous One Solution Cell Proliferation Assay (CellTiter 96; PROMEGA, Fitchburg, United States) was added. After 2 h incubation at 37^°^C, the absorbance of samples was recorded at 490 nm (TECAN, Infinita M200, Austria). A standard toxicological parameter IC_50_ was calculated by non-linear regression from a semi-logarithmic plot of incubation concentration vs. percentage of absorbance relative to untreated controls using GraphPad Prism 9 software.

Results of the experiments are presented as inhibitory concentration, which reduces the viability of the cell population to 50% from the maximal viability (IC_50_). The IC_50_ values were calculated in GraphPad Prism for tested compounds in the concentration range 1–250 μM.

### Evaluation of *in vivo* toxicity using the animal model, *Galleria mellonella*

For the purpose of *in vivo* toxicity assessment, an invertebrate model using the larvae of *Galleria mellonella* (*G. mellonella*) was included. *G. mellonella* animals were reared in the laboratory of the Department of Microbiology and Immunology, at the Faculty of Pharmacy in Hradec Králové, Charles University. The larvae were fed with an artificial diet in accordance with Haydakm (1936) and reared in the dark at 29^°^C. Only fully vital, cream-colored larvae with weight ranging from 250 to 300 mg were selected for tested compounds administration before each experiment.

In brief, the tested compounds were dissolved in DMSO and diluted to the required working concentration using a phosphate-buffer saline, pH 7.4 (Sigma-Aldrich, United States), where the final concentration of DMSO corresponded to 30% (v/v). The samples were administered into the larvae at a total volume of 10 μl/per larva, into hemocoel through the last left proleg using a Hamilton syringe. In each experiment, two control groups (untreated control, buffer-injected control) were included. The inoculated larvae and control groups were then incubated in Petri dishes at 37°C. The survival and conditions of larvae were recorded over a 120-h period (24, 48, and 120-h after administration). Death was defined as the complete loss of mobility, including a physical stimulus using a plastic pipette. The mortality for each dose/time interval was calculated, and survival curves were designed *via* SW analysis (GraphPad Prism 9.0.0, United States).

### Statistical analysis

Screening survival experiments within *in vivo* toxicity studies consisted of 53 individuals for evaluation of CH-0y (group one, *n* = 4; all other groups, *n* = 7 per group), and 53 individuals for CH-0w (group one, *n* = 5; group five, *n* = 6; all other groups, *n* = 7 per group). All data outputs were analyzed by GraphPad Prism software version 9.0.0 (United States). Data from survival experiments were subjected to the log-rank Mantel-Cox (curve comparison) test. Data from the evaluation of PAE were subjected to a one-way analysis of variance (ANOVA) and direct group-group comparison. Results were considered significant at a *p*-value < 0.05 in all analyses.

## Results and discussion

### Searching for and characterizing new perspective anti-infectives among chalcone-based derivatives

**FIGURE 1:**
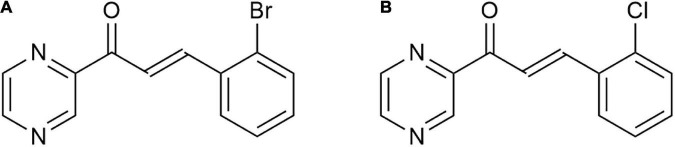
The structures of the studied compounds CH-0y **(A)**, and CH-0w **(B)**, created using ChemDraw 20.0 (PerkinElmer).

The antimicrobial activity of chalcones has been in our focus of research for a long time. This research started with the synthesis of hydroxylated, methoxylated, and aminated pyrazine-based chalcones (Opletalova et al., 2002), and continued with the synthesis of the same structural pattern with nitro (Opletalova et al., 2006) and halogen (Chlupacova et al., 2005; Kucerova-Chlupacova et al., 2015, 2016) substitutions. As for the antibacterial activity, the presence of an electron-withdrawing group in the ring B was highlighted in our last study (Kucerova-Chlupacova et al., 2016). According to a re-evaluation of antibacterial activity against an extended panel of bacterial collection strains, two perspective compounds, CH-0y (Figure 1A) and CH-0w (Figure 1B), were selected after careful consideration for this detailed and comprehensive characterization of the *in vitro* antibacterial effects and *in vivo* toxicity. Before evaluating different aspects of compound vs. bacteria interaction *in vitro*, the identity and purity of the compounds were checked. The data obtained from NMR, MS, and EA are presented below.

#### CH-0y

^1^H NMR δ 9.38 (d, J = 1.5, 1H), 8.78 (d, J = 2.4, 1H), 8.68 (dd, J = 2.4, 1.5, 1H), 8.35 (d, J = 15.9, 1H), 8.11 (d, J = 15.9, 1H), 7.85 (dd, J = 7.8, 1.7, 1H), 7.64 (dd, J = 8.0, 1.3, 1H), 7.36 (m, 1H), 7.29 – 7.23 (m, 1H). ^13^C NMR δ 188.3, 148.3, 147.5, 144.9, 143.9, 143.3, 134.8, 133.6, 131.7, 128.1, 127.7, 126.5, 122.7.

MS: [M + H]^+^ = 289.0 (calculated exact mass 289.00).

EA: calcd. for C_13_H_9_BrN_2_O (Mr 289.13) 54.00% C; 3.14% H; 9.69% N, found 54.09% C; 2.86% H; 9.63% N.

#### CH-0w

^1^H NMR δ 9.38 (d, J = 1.5, 1H), 8.78 (d, J = 2.4, 1H), 8.68 (dd, J = 2.4, 1.5, 1H), 8.39 (d, J = 16.0, 1H), 8.15 (d, J = 16.0, 1H), 7.87 (dd, J = 7.6, 1.9, 1H), 7.44 (dd, J = 7.8, 1.5, 1H), 7.33 (m, 2H).^13^C NMR δ 188.3, 148.3, 147.5, 144.9, 143.3, 141.3, 136.0, 133.0, 131.6, 130.3, 128.0, 127.1, 122.4.

MS: [M + H]^+^ = 245.3 (calculated exact mass 245.05); [2M-H]^–^ = 487.1 (calculated exact mass 487.07).

EA: calcd. for C_13_H_9_ClN_2_O (Mr 244.68) 63.82% C; 3.71% H; 11.45% N, found 63.32% C; 3.48% H; 11.41% N.

Both compounds meet the Lipinski criteria applied in basic drug design in medicinal chemistry, i.e., the compounds have good membrane permeability, if log *P* ≤ 5, the number of hydrogen bond acceptors ≤ 10, molecular weight ≤ 500, and the number of hydrogen bond donor ≤ 5 (Lipinski et al., 1997), and the general requirement for chalcones with one ring being more hydrophilic and the other one being more lipophilic. Halogen substitution can support halogen binding to the potential target in bacteria (Hamilton et al., 2021), especially in the case of brominated CH-0y. The fluorinated analog exerted a weaker antibacterial effect, as well as the other derivatives which is in accordance with the structure-activity results of a comprehensive review on antibacterial activity of chalcones (Kucerova-Chlupacova et al., 2016; Dan and Dai, 2020).

### Recognition of the anti-staphylococcal and anti-enterococcal potential/activity of CH-0y and CH-0w

CH-0y and CH-0w were subsequently subjected to an evaluation of their antibacterial activity *in vitro* using an extended panel of selected microbial strains from the group of gram-positive bacteria, including both reference strains, registered in collections of microorganisms and clinical isolates with a determined susceptibility/resistance profile (Supplementary Table 1 and Supplementary Figures 2, 3). In summary, four reference strains and twelve clinical isolates were included in the antibacterial activity evaluation (Table 1). For the compound CH-0y, the antibacterial activity (expressed as MIC) against staphylococci was registered in the concentration range from 15.625 to 62.5 μM (incubation for 24 h). The highest activity of CH-0y was revealed against some CoNS clinical isolates, namely, *S. epidermidis* and *S. lugdunensis.* Inconsistent results were obtained for the activity of this compound against the included *Enterococcus* spp. strains. In the reference strain, *Enterococcus faecalis* (*E. faecalis*, ATCC 29212), no activity was registered within the tested concentration range. On the contrary, the activity expressed as MIC, corresponding to a concentration range of 31.25–62.5 μM (Table 1), was registered in all three included *E. faecium* clinical isolates, recognized as MDR (Supplementary Table 1). This finding might not be so surprising. As indicated in the study conducted by Quiloan et al. (2012), *E. faecalis* can be distinguished from *E. faecium via* its differential susceptibility to antimicrobial compounds. Thus, in these two mentioned species of the genus *Enterococcus*, the same susceptibility to tested compounds cannot be assumed. In addition, although it is stated that while *E. faecalis* is associated with increased virulence, *E. faecium* very often shows signs of multidrug resistance, which has a significant impact on the course of antimicrobial therapy (Krawczyk et al., 2020).

**TABLE 1:** Antibacterial activity of CH-0y and CH-0w against reference strains and clinical isolates of the genus *Staphylococcus* and *Enterococcus*.

Bacterial strain (ATCC, ID No.)	CH-0y	CH-0w
	
	MIC (μM) 24 h/48 h	
*Staphylococcus aureus* subsp. *aureus*, ATCC 29213	31.25/125	31.25/125
*Staphylococcus aureus* subsp. *aureus*, MRSA, ATCC 43300	31.25/62.5	31.25/62.5
*Enterococcus faecalis*, ATCC 29212	>500/>500	62.5/250
*Staphylococcus aureus*, VRSA, CCM 1767	62.5/ND	62.5–125/ND
*Staphylococcus aureus*, VRSA, 203/16 NIPH	62.5/ND	125/ND
*Staphylococcus aureus*, MRSA, 131/16	62.5/62.5	62.5/62.5
*Staphylococcus aureus*, 136/16	31.25/62.5	31.25/62.5
*Staphylococcus aureus*, 137/16	31.25/62.5	31.25/62.5
*Staphylococcus epidermidis*, 196/16	31.25/31.25	31.25/31.25
*Enterococcus faecium*, VRE, 198/16	31.25/31.25	62.5/ > 125
*Staphylococcus lugdunensis*, 1/21	15.625/ND	62.5/ND
*Staphylococcus epidermidis*, 3/21	15.625/ND	62.5/ND
*Staphylococcus epidermidis*, 4/21	31.25/ND	31.25/ND
*Staphylococcus epidermidis*, 5/21	15.625/ND	62.5/ND
*Enterococcus faecium*, VRE, 17/21	62.5/ND	62.5/ND
*Enterococcus faecium*, VRE, 18/21	62.5/ND	62.5/ND

Antibacterial action was evaluated by the microdilution method according to EUCAST recommendations, with slight modifications. Minimum inhibitory concentration (MIC) was evaluated after 24 and 48 h of cultivation by visual inspection and spectrophotometric measurement.

ATCC, American Type Culture Collection; ID No., internal laboratory identification number; MRSA, methicillin-resistant *Staphylococcus aureus*; VRSA, vancomycin-resistant *Staphylococcus aureus*; VRE, vancomycin-resistant *Enterococcus* sp.; NIPH, The National Institute of Public Health in Prague; Czech Republic; ND, not determined.

The compound CH-0w showed anti-staphylococcal activity in the range of tested concentration from 31.2 to 125 μM. The lowest activity (125 μM) was registered in two included VRSA strains. For this compound, in contrast to the previous one, a uniform anti-enterococcal activity (62.5 μM) was registered in both *E. faecalis* and *E. faecium* strains.

Chalcones rightly attract attention and are inspiring structures within the field of medicinal chemistry. The evidence of this fact is supported by the availability of a wide range of studies dealing with the synthesis and biological evaluations of various groups of synthetic chalcone-derived structures. In the field of anti-infective drug research, a number of research studies demonstrating promising activities against staphylococci in chalcone-derived compounds with halogen substitution have been published. Namely, the studies were focused on chalcone derivatives bearing the 2,4-thiazolidinedione and benzoic acid moieties (MIC activity ranging from 1 to > 64, μg/mL) (Liu et al., 2011), chalcone derivates containing a rhodanine-3-acetic acid moiety (activity ranging from 2 to > 64 μg/mL) (Chen et al., 2010), chalcones with aliphatic amines in ring A (activity ranging from 20 to 300 μM) (Nielsen et al., 2005), substituted oxathiolone-fused chalcones (MIC activity ranging from 7.8 to > 1,000 μg/mL) (Nielsen et al., 2004; Bowman et al., 2007; Konieczny et al., 2007; Jin et al., 2012; Hamada and Abdo, 2015). Some of these compounds have been patented so far (Matos et al., 2015).

Similarly, certain anti-enterococcal activities have been recognized in various series of chalcone derivatives, such as hydroxylated chalcones (Feng et al., 2014), 1,2,3-triazole-linked chalcones (Kant et al., 2016), and other chalcone derivatives (Liaras et al., 2011; Burmaoglu et al., 2017; Zhang et al., 2018). When focusing on halogenated derivatives, it is important to mention the group of thiazole-based chalcones, with activity against *E. faecalis* corresponding to MIC = 76.4 mM, which was even better than the one of ampicillin.

### CH-0y and CH-0w show a bactericidal mode of action against staphylococci

The microdilution broth method was employed to recognize the bacteriostatic *vs.* bactericidal action. Two SA and one MRSA strains were exposed for 24 h to CH-0y and CH-0w at various concentrations, and subsequently, the spread plate technique for calculating CFU was employed. As presented in Table 2, after exposure of these strains to CH-0y and CH-0w at a concentration corresponding to 4 × MIC, the percentage reduction in the number of CFU/mL compared to the initial inoculum corresponded to ≥ 99.398–99.957%. According to the mentioned criterion (section “Detection of the bacteriostatic vs. bactericidal mode of action”), it can be concluded that both CH-0y and CH-0w represent compounds with bactericidal action, with more unambiguous results being obtained for CH-0y. The bactericidal action is also partially indicated by the MIC values (Table 1) evaluated after both 24 h and further after 48 h of incubation. No shift or a one-step shift may point out a bactericidal mode of action. The bactericidal mode of action was recognized in some natural chalcones, such as xanthohumol and chalcone-based derivatives (Ahmed et al., 2019; Bocquet et al., 2019; Xu et al., 2019; Coskun et al., 2021). Among halogenated chalcone derivatives, the bactericidal action against SA, or MRSA has been explored in fluorinated chalcone-1,2,3-triazole hybrids, or chlorinated benzofuran chalcones (Xu et al., 2019; Coskun et al., 2021).

**TABLE 2:** Reduction of the staphylococcal viability after 24-h exposure to 4-fold of a minimum inhibitory concentration of CH-0y and CH-0w.

Bacterial strain (ID No.)	CH-0y	
	c (μM)/4-fold of MIC	% of reduction*
*Staphylococcus aureus*, MRSA, 131/16	125	99.892
*Staphylococcus aureus*, 136/16	250	99.957
*Staphylococcus aureus*, 137/16	125	99.952

	**CH-0w**	
	**c (μM)/4-fold of MIC**	% of reduction*
	
*Staphylococcus aureus*, MRSA, 131/16	125	99.8
*Staphylococcus aureus*, 136/16	250	99.889
*Staphylococcus aureus*, 137/16	125	99.398

The microdilution broth method and spread plate technique for calculating colony-forming units were employed in the evaluation.

MIC, minimum inhibitory concentration; MRSA, methicillin-resistant *Staphylococcus aureus*.

*Reduction in the number of colony-forming units (CFU) compared to CFU number of initial bacterial inoculum.

### The anti-staphylococcal action of CH-0y and CH-0w in combination with selected conventional antibiotic drugs

For the checkerboard studies, the anti-staphylococcal/antibacterial drugs, namely, VAN, SXT, RIF, CIP, and LIN, were included for partner drug combination with CH-0y and CH-0w. Antibiotic drugs with different mechanisms of action were preferentially and purposefully selected for these assays. According to the FICI calculation, it was revealed that none of the above-mentioned compounds showed a synergistic interaction in combination with both CH-0y and CH-0w compounds (Supplementary Tables 2, 3 and Supplementary Figures 4, 5). The additive effect was revealed only in a combination of SXT + CH-0y in some concentration ratios. On the other hand, it is necessary to point out that in some concentration ratios of combination SXT + CH-0y, an antagonistic effect was revealed, as well. The combination of SXT + CH-0w revealed the antagonistic effect in one concentration ratio. In others, an indifferent effect was detected. In combination of VAN, CIP, and LIN with CH-0y or CH-0w, the same indifferent effect was revealed in all concentration ratios. In some concentration ratios, the interaction of RIF + CH-0y has led to decreased activity, evaluated as an antagonistic effect. Different results have been achieved in a combination of RIF + CH-0w. In all tested concentration ratios, the indifferent effect was determined.

Generally, compounds demonstrating only weak antimicrobial activity can in combination with some antibiotics contribute to an increase in total antibacterial activity, and likewise, they can be also helpful in restoring the antimicrobial effect of separate invalid antibiotics.

The strategy to potentiate the antibacterial activity *via* a combination of chalcones with commercially available antibiotics has also been demonstrated in the studies of the biological activities of a number of chalcones, such as xanthohumol (Bocquet et al., 2019), 1,3-bis-(2-hydroxyphenyl)propenone (Božić et al., 2014), or prenylated chalcone isolated from the roots of *Sophora flavescens* (Lee et al., 2010).

In summary, although the synergistic effect of a combination of two or more compounds within drug combination therapy is strongly preferred (an indication of reduced toxicity and an adverse effect due to reduced dosage) (Kumar et al., 2012), additive and indifferent effects can also be seen as beneficial. It should be kept in mind that one option to prevent the risk of resistance development and the spread of resistance also lies in combination therapy. Within this therapy, especially antimicrobials that target different molecular structures or multi-subunit macromolecular machineries are preferably selected.

### A study of the post-antimicrobial and post-antimicrobial sub-MIC effects of CH-0y and CH-0w, and a comparison with two conventional antimicrobial drugs, ciprofloxacin and vancomycin

PAE represents the potential to suppress bacterial growth after brief exposure to antibacterial drugs (Spangler et al., 1998, Chen et al., 2018). This important parameter of antibiotic action is widely used as a predictor of pharmacodynamic activity and is recommended in the pre-clinical evaluation of all candidate antimicrobial compounds (Stubbings et al., 2004). PAE and PAE-SME are factors that influence especially the optimal antimicrobial drug dosing interval. Generally, drugs with no PAE require more frequent administration than those that demonstrate PAE (Spangler et al., 1998). For evaluating these parameters, a new method using a spectrophotometric procedure was involved. This methodical approach brings many advantages compared to the traditionally used method involving the determination of viable cell numbers (Löwdin et al., 1993). In this methodical arrangement, PAE and PAE-SME are defined as the difference in the time required for the exposed and unexposed bacterial cultures to grow to a chosen cut-off point on the absorbance curve (mainly corresponding to the middle or late exponential phase of the growth of the unexposed, positive growth control). It has been suggested that an alteration of DNA is possibly responsible for PAE (Spangler et al., 1998). Based on this hypothesis, from the included compounds, the conventional antimicrobial drug ciprofloxacin, inhibiting DNA replication, should show one of the most promising, long-lasting PAEs. As shown in Table 3 and Supplementary Figure 6, after 2 h of exposure of the MRSA strain, ATCC 43300, to the 2 × MIC of ciprofloxacin, the time of cultivation to reach the optical density 0.2 (540 nm) of bacterial suspension compared to positive growth control (untreated bacterial cells) reached 9.28 × multiplicity. Conclusively, after a short exposure to 2 × MIC of the included compounds, only this antibacterial drug showed the longest PAE. However, such a conclusion cannot be made for a short exposure of MRSA at 1 × MIC, where the largest effect was noted for application of the chalcones, CH-0y and CH-0w. Remarkably, at 2 × MIC, the effect of the chalcones remained about the same, while the effect for vancomycin doubled and for ciprofloxacin quadrupled. The statistical analysis revealed that, unlike CH-0y, CH-0w shows a statistically significant difference in time growth delay compared to the reference antibiotic drug, ciprofloxacin (Supplementary Figure 7). Therefore, it can be concluded that after 2 × MIC exposure, the CH-0y comparably contributes to the time growth delay with ciprofloxacin (no significant difference was recognized between CH-0y and ciprofloxacin). However, after 1 × MIC exposure, no significant difference in contribution to time growth delay was noted in both CH-0y and CH-0w compared to ciprofloxacin (Supplementary Figure 8).

**TABLE 3:** Post-antimicrobial effect presented as a time delay in the growth of a bacterial strain after 2 h of exposure to CH-0y, CH-0w, and the conventional drugs, vancomycin, and ciprofloxacin.

	Compound	Time (minutes)	Multiplicity*
**Exposition to 1 × MIC**			
	Vancomycin	179	1.77
	Ciprofloxacin	184	1.82
	CH-0y	243	2.41
	CH-0w	219	2.17
	Positive growth control	101	1
**Exposition to 2 × MIC**			
	Vancomycin	407	4.02
	Ciprofloxacin	937	9.28
	CH-0y	265	2.62
	CH-0w	256	2.53
	Positive growth control	101	1

The bacterial strain, *Staphylococcus aureus* (MRSA), ATCC 43300 was exposed to concentrations of the included compounds, corresponding to 1 ×, or 2 × MIC (minimum inhibitory concentration). For evaluating the time delay, a cut-off value corresponding to O.D. 0.2 was used.

*Multiplicity of time for achievement of the cut off O.D. value in untreated bacterial growth control.

An independent experimental procedure for the study of PAE-SME effects revealed that ciprofloxacin showed the highest time delay in the bacterial growth (the longest PAE) in all sub-inhibitory concentrations (Table 4 and Supplementary Figure 9). The PAE-SME of CH-0y and CH-0w was lower compared to both conventional drugs, but not negligible. The potential to suppress bacterial growth within the action of sub-MIC concentrations of CH-0y and CH-0w seems to be almost comparable. The role of chalcone derivatives in delaying bacterial growth represented as PAE was explored in another comprehensive study focused on halogenated chalcone derivatives (isoliquiritigenin derivatives). Similarly, the beneficial impact of these derivatives on bacterial growth delay was revealed (Gupta et al., 2019).

**TABLE 4:** Post-antimicrobial sub-MIC effect presented as a time delay in the growth of a bacterial strain after 2 h of exposure to CH-0y, CH-0w, and the conventional drugs, vancomycin, and ciprofloxacin in concentrations corresponding to 1 × MIC, with subsequent cultivation in a medium with sub-inhibitory concentrations of the included compounds.

	Sub-inhibitory concentration			
	0.8 × MIC	0.6 × MIC	0.4 × MIC	0.2 × MIC
	Time (minutes)			
**Compound**				
Vancomycin	>1,500	>1,500	701	637
Ciprofloxacin	>1,500	>1,500	>1,500	1,197
CH-0y	913	702	488	358
CH-0w	1,018	830	532	353
Multiplicity*				
Vancomycin	>6.25	>6.25	2.921	2.65
Ciprofloxacin	>6.25	>6.25	>6.25	4.99
CH-0y	3.80	2.93	2.03	1.49
CH-0w	4.24	3.46	2.22	1.47

The bacterial strain, *Staphylococcus aureus* (MRSA), ATCC 43300, and subinhibitory concentrations corresponding to 0.8, 0.6, 0.4, 0.2 × MIC were employed in the assay. For evaluating the time delay, a cut-off value corresponding to O.D. 0.2 was used.

*Multiplicity of time for achievement of the cut-off O.D. value in untreated bacterial growth control.

### Focus on the *in vitro* cytotoxicity and the *in vivo* toxicity of CH-0y and CH-0w

*In vitro* cytotoxicity of the tested compounds was measured using the human kidney epithelial cell line HK-2, and the parameter IC_50_ was used as the indicator of cytotoxicity. As shown in Supplementary Figure 10, the determined IC_50_ values indicate relatively higher cytotoxicity of CH-0w (122.9 μM) in comparison with CH-0y (143.3 μM). The selectivity index (SI) of compounds, calculated as the ratio of IC_50_/MIC, can be a representative parameter for the expression of the compound’s efficacy *in vitro* and can help with the recognition of potential candidates for future drugs. Compounds with SI above 10 are considered great drug candidates since they present sufficient effectiveness and safety. The CH-0y has shown the range of SI = 9.2–2.3, and for CH-0w, the range of SI = 4–1 has been recognized. So, inferior results were achieved obviously in CH-0w. However, the mutual interaction, adjuvant and anti-infective drug, should be reflected when considering compounds as suitable adjuvants to existing antimicrobial therapy. The basic principle of combination therapy or the introduction of adjuvants is the reduction of therapeutic doses and, therefore, reduction of toxicity. In addition, the reduction of the risk of the development and spread of resistance is achieved by this therapeutical strategy, as well.

In experimental settings, the *in vivo* evaluation of toxicity has been performed, as well. The greater emphasis should be generally aimed at results from this evaluation. For the purpose an *in vivo* toxicity assessment, an invertebrate model using the larvae of *Galleria mellonella* was included. *Galleria mellonella* represents an alternative animal model that is increasingly being used as a replacement for mice and rats in infection and toxicity studies (Junqueira, 2012; Ignasiak and Maxwell, 2017). The use of this model in toxicity testing brings several benefits and seems to be fully convenient for the discrimination of toxic and non-toxic chemicals (Allegra et al., 2018).

**FIGURE 2:**
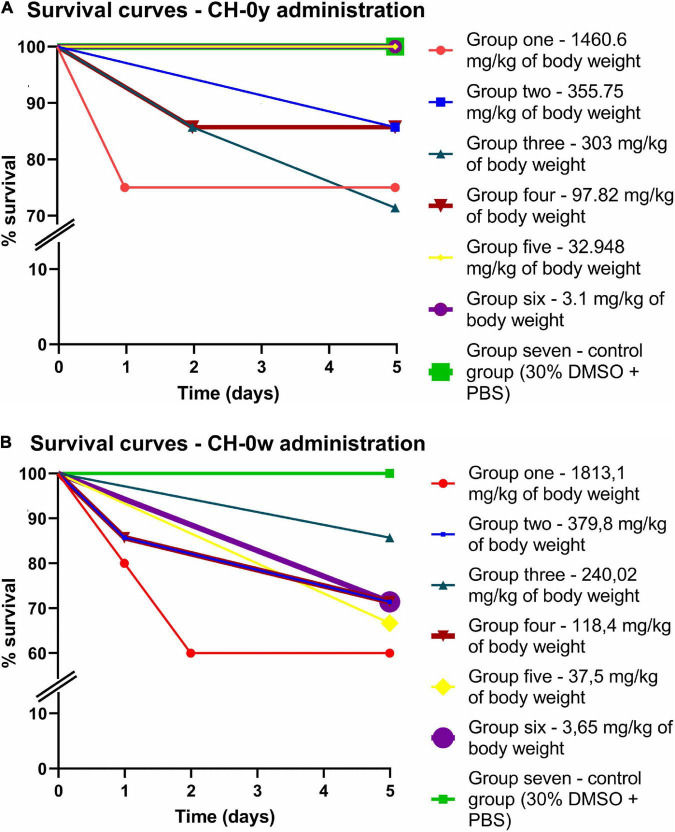
Survival curves of animal model, *Galleria mellonella*, after intra-hemocoel administration of CH-0y **(A)** and CH-0w **(B)**. After administration, larvae were incubated at 37^°^C for 5 days and inspected after 24, 48, and 120 h of incubation.

The larvae were divided into groups according to the final amount of administered compound per kg of the animal’s body weight. The doses approximately corresponded to the recommendation of OECD (Organization for Economic Co-operation and Development) test guidelines for chemicals. The tested compounds were administered to animals *via* the intra-hemocoel route through the last left proleg. It can be stated that this administration method into hemocoel mimics the intravenous route of administration in mammals and reflects systemic toxicity. As shown in Figures 2A,B and Supplementary Table 4, in both tested compounds and all included groups, LD_50_ (median lethal dose leading to the death of 50% of the animals) was not reached. Therefore, it can be concluded that the LD_50_ of CH-0y corresponds to a dose > 1460.6 mg/kg of body weight of larvae, and the LD_50_ of CH-0w corresponds to a dose > 1813.1 mg/kg of body weight of larvae. In CH-0y, the highest mortality (25%) was registered in group one, corresponding to a mean dose of 1460.6 mg/kg of body weight. For CH-0w, the highest mortality rate (40%) was recognized in the group with the highest administered mean dose, 1813.1 mg/kg of body weight. However, surprisingly, relatively high mortality, 33.3% and 28.571% was recognized in the fifth and sixth groups (mean doses of 37.5 and 3.65 mg/kg of body weight, respectively). The unexpected rate of mortality in these groups might be related to traumatic inoculation (the mortality was registered at a time interval of 120 h after administration), but it also might have been skewed by the smaller number of animals in the groups, or it might also reflect different compounds’ bioavailability in these concentration ranges. No significant effect of CH-0y and CH-0w compounds on the survival of larvae was registered after data analysis by the log-rank Mantel-Cox curve comparison test [CH-0y: χ^2^(6) = 5.896, *P* = 0.4349, Figure 2A; CH-0w: χ^2^(6) = 4.220, *P* = 0.6469, Figure 2B). For CH-0y, hazard ratios (Mantel-Haenszel pairwise comparison test) calculated from the increases in CH-0y from 97.82 to 303, 355.75, and 1460.6 mg/kg of body weight, corresponding to 2.019 (95% CI, 0.1977–20.62), 0.9257 (95% CI, 0.05766–14.86), and 2.143 (95% CI, 0.1121–40.94). Hazard ratios for CH-0w, calculated from the increase 37.5 to 118.4, 379.8, and 1813.1 mg/kg of body weight, correspond to 1.142 (95% CI, 0.398–9.320), 1.142 (95% CI, 0.1398–9.320), and 2.077 (95% CI 0.2472–17.46), see Supplementary Tables 5, 6 for all pairwise comparisons. Based on these findings, both CH-0y and CH-0w can be categorized into the GHS (Globally Harmonized System) class 4, which represents non/low toxic compounds (Ignasiak and Maxwell, 2017). In summary, as it was recognized within *in vivo* toxicity testing, CH-0y seems to be a more promising compound in relation to possible toxic adverse effects.

## Conclusion

Chalcones and their derivatives are considered highly attractive structures in medicinal chemistry, especially regarding their wide range of biological activities. Antimicrobial activity has been identified, which encourages their inclusion as opportune candidate compounds to combat the global crisis of antimicrobial resistance.

In this study, we focused on performing an extensive and comprehensive analysis to describe in more detail the candidate compound-microbe interaction and to recognize “friendly” attributes for their use as possible drugs.

Two pyrazine-based halogenated chalcones with recognized anti-infective potential against gram-positive bacteria were includedxs in the evaluation. The first one with the 2-bromo substitution in the ring B (CH-0y), and the second one with the 2-chloro substitution in the ring B (CH-0w).

When comparing anti-staphylococcal activities, more promising results were obtained with CH-0y, where the highest activity against coagulase-negative *S. epidermidis* and *S. lugdunensis* was detected. In CH-0y and CH-0w, the activity (MIC = 62.5 μM) against the clinical isolate strains of *Enterococcus faecium* with significant multidrug resistance profiles was also revealed. Similarly, both compounds within the tested concentration ranges were active against medically highly significant, methicillin- and vancomycin-resistant strains. Both CH-0y and CH-0w showed a bactericidal mode of action, which can be considered advantageous in the fight against infectious agents. Unfortunately, within the checkerboard studies, no synergistic effect of CH-0y or CH-0w with five selected conventional antibiotics was revealed. On the other hand, in some concentration combinations (CH-0y together with trimethoprim/sulfamethoxazole), additive effects were shown. Nevertheless, the indifferent effect recognized in many other combinations does not limit the potential use of these compounds in possible combination therapy. In addition, within PAE and PAE-SME studies, CH-0y and CH-0w showed a non-negligible impact on time delay in the bacterial growth recovery, which can also be considered a promising pharmacodynamic parameter. According to the study of cytotoxicity with the inclusion of non-tumor cell line and the expression of a compound’s efficacy *in vitro* using the parameter, SI, a better, however, not fully desirable outputs were achieved for the compound, CH-0y. On the other hand, within the alternative animal model, *Galleria mellonella*, only the non/low toxic potential of both compounds, CH-0y and CH-0w, was recognized.

In conclusion, within the increased prevalence of infections caused by MDR staphylococcal and enterococcal pathogens, it would be entirely unjustified to overlook these compounds at least as possible adjuvants to selected antibiotic drugs.

## Data availability statement

All datasets generated for this study are included in the article/Supplementary material. Requests to access the raw data supporting the conclusions of this article should be directed to the corresponding authors.

## Author contributions

KK contributed in designing, directing, and performing microbiological and *in vivo* toxicological studies. MK-C contributed to the design, synthesis, and characterization of halogenated pyrazine-based chalcones and directed the chemical part of the study. KK and OJ carried out the basic antibacterial screening. AD contributed to checkerboard assays. BV and MV carried out identification and susceptibility profile evaluation of clinical isolates. PH carried out PAE evaluations. PB, JM, KK, and FT carried out toxicity studies. KK and PH performed the statistical analysis. KK and MK-C wrote the manuscript. All authors read and approved the final manuscript.

## References

[B1] Abouelhassan Y. Garrison A. T. Yang H. Chávez-Riveros A. Burch G. M. Huigens R. W. III (2019). Recent progress in natural-product-inspired programs aimed to address antibiotic resistance and tolerance. *J. Med. Chem.* 62 7618–7642. 10.1021/acs.jmedchem.9b00370 30951303 PMC6742553

[B2] Ahmed M. O. Baptiste K. E. (2018). Vancomycin-resistant enterococci: A review of antimicrobial resistance mechanisms and perspectives of human and animal health. *Microb. Drug Resist.* 24 590–606. 10.1089/mdr.2017.0147 29058560

[B3] Ahmed N. Konduru N. K. Owais M. (2019). Design, synthesis and antimicrobial activities of novel ferrocenyl and organic chalcone based sulfones and bis-sulfones. *Arab. J. Chem.* 12 1879–1894. 10.1016/j.arabjc.2014.12.008

[B4] Allegra E. Titball R. W. Carter J. Champion O. L. (2018). *Galleria mellonella* larvae allow the discrimination of toxic and non-toxic chemicals. *Chemosphere* 198 469–472. 10.1016/j.chemosphere.2018.01.175 29425947

[B5] Barsoumian A. E. Mende K. Sanchez C. J. Jr. Beckius M. L. Wenke J. C. Murray C. K. (2015). Clinical infectious outcomes associated with biofilm-related bacterial infections: A retrospective chart review. *BMC Infect. Dis.* 15:223. 10.1186/s12879-015-0972-2 PMC4458033 26049931

[B6] Battenberg O. A. Yang Y. Verhelst S. H. Sieber S. A. (2013). Target profiling of 4-hydroxyderricin in *S. aureus* reveals seryl-tRNA synthetase binding and inhibition by covalent modification. *Mol. Biosyst.* 9 343–351. 10.1039/c2mb25446h 23295910

[B7] Becker K. Heilmann C. Peters G. (2014). Coagulase-negative staphylococci. *Clin. Microbiol. Rev.* 27 870–926. 10.1128/cmr.00109-13 25278577 PMC4187637

[B8] Bender J. K. Cattoir V. Hegstad K. Sadowy E. Coque T. M. Westh H. (2018). Update on prevalence and mechanisms of resistance to linezolid, tigecycline and daptomycin in enterococci in Europe: Towards a common nomenclature. *Drug Resist. Updat.* 40 25–39. 10.1016/j.drup.2018.10.002 30447411

[B9] Bocquet L. Sahpaz S. Bonneau N. Beaufay C. Mahieux S. Samaillie J. (2019). Phenolic compounds from *Humulus lupulus* as natural antimicrobial products: New weapons in the fight against methicillin resistant *Staphylococcus aureus*, *Leishmania mexicana* and *Trypanosoma brucei* strains. *Molecules* 24:1024. 10.3390/molecules24061024 30875854 PMC6472001

[B10] Bowman M. D. O’Neill J. C. Stringer J. R. Blackwell H. E. (2007). Rapid identification of antibacterial agents effective against *Staphylococcus aureus* using small-molecule macroarrays. *Chem. Biol.* 14 351–357. 17462570 10.1016/j.chembiol.2007.03.006

[B11] Božić D. D. Milenković M. Ivković B. Ćirković I. (2014). Antibacterial activity of three newly-synthesized chalcones & synergism with antibiotics against clinical isolates of methicillin-resistant *Staphylococcus aureus*. *Indian J. Med. Res.* 140 130–137. 25222788 PMC4181146

[B12] Burmaoglu S. Algul O. Gobek A. Aktas Anil D. Ulger M. Erturk B. G. (2017). Design of potent fluoro-substituted chalcones as antimicrobial agents. *J. Enzyme Inhib. Med. Chem.* 32 490–495. 10.1080/14756366.2016.1265517 28118738 PMC6010113

[B13] Chambers H. F. Deleo F. R. (2009). Waves of resistance: *Staphylococcus aureus* in the antibiotic era. *Nat. Rev. Microbiol.* 7 629–641. 19680247 10.1038/nrmicro2200 PMC2871281

[B14] Chen H. Li L. Liu Y. Wu M. Xu S. Zhang G. (2018). In vitro activity and post-antibiotic effects of linezolid in combination with fosfomycin against clinical isolates of *Staphylococcus aureus*. *Infect. Drug Resist.* 11 2107–2115. 10.2147/idr.s175978 30464553 PMC6219420

[B15] Chen Z. H. Zheng C. J. Sun L. P. Piao H. R. (2010). Synthesis of new chalcone derivatives containing a rhodanine-3-acetic acid moiety with potential antibacterial activity. *Eur. J. Med. Chem.* 45 5739–5743. 10.1016/j.ejmech.2010.09.031 20889240

[B16] Chlupacova M. Opletalova V. Kunes J. Silva L. Buchta V. Duskova L. (2005). Synthesis and biological evaluation of some ring-substituted (E)-3-aryl-1-pyrazin-2-ylprop-2-en-1-ones. *Folia Pharm. Univ. Carol.* 33, 31–43.

[B17] Coskun D. Dalkilic S. Dalkilic L. K. Coskun M. F. (2021). Synthesis, characterization, and antimicrobial potential of some chlorinated benzofuran chalcones. *Prog. Nutr.* 23:e2021256. 10.23751/pn.v23iS2.11963

[B18] Dan W. Dai J. (2020). Recent development of chalcones as potential antibacterial agents in medicinal chemistry. *Eur. J. Med. Chem.* 187:111980. 10.1016/j.ejmech.2019.111980 31877539

[B19] Elkhalifa D. Al-Hashimi I. Al Moustafa A. E. Khalil A. (2021). A comprehensive review on the antiviral activities of chalcones. *J. Drug Target.* 29 403–419. 10.1080/1061186x.2020.1853759 33232192

[B20] European Committee for Antimicrobial Susceptibility Testing [EUCAST] of the European Society for Clinical Microbiology Infectious Diseases [ESCMID]. (2003). Eucast discussion document E. Dis 5.1: Determination of Minimum Inhibitory Concentrations (MICs) of antibacterial agents by broth dilution. *Clin. Microbiol. Infect.* 9 9–15. 10.1046/j.1469-0691.2003.00790.x

[B21] Feng L. Maddox M. M. Alam M. Z. Tsutsumi L. S. Narula G. Bruhn D. F. (2014). Synthesis, structure–activity relationship studies, and antibacterial evaluation of 4-chromanones and chalcones, as well as olympicin a and derivatives. *J. Med. Chem.* 57 8398–8420. 10.1021/jm500853v 25238443 PMC4207537

[B22] Friedman N. D. Temkin E. Carmeli Y. (2016). The negative impact of antibiotic resistance. *Clin. Microbiol. Infect.* 22 416–422. 10.1016/j.cmi.2015.12.002 26706614

[B23] Fukai T. Marumo A. Kaitou K. Kanda T. Terada S. Nomura T. (2002). Antimicrobial activity of licorice flavonoids against methicillin-resistant *Staphylococcus aureus*. *Fitoterapia* 73 536–539. 10.1016/s0367-326x00168-5 12385884

[B24] Gupta V. K. Gaur R. Sharma A. Akther J. Saini M. Bhakuni R. S. (2019). A novel bi-functional chalcone inhibits multidrug resistant *Staphylococcus aureus* and potentiates the activity of fluoroquinolones. *Bioorg. Chem.* 83 214–225. 10.1016/j.bioorg.2018.10.024 30380450

[B25] Hamada N. M. Abdo N. Y. (2015). Synthesis, characterization, antimicrobial screening and free-radical scavenging activity of some novel substituted pyrazoles. *Molecules* 20 10468–10486. 10.3390/molecules200610468 26060913 PMC6272688

[B26] Hamilton V. Harris C. Hall C. L. Potticary J. Cremeens M. E. D’Ambruoso G. D. (2021). Structural effects of halogen bonding in iodochalcones. *Acta Crystallogr. B Struct. Sci.* 77 347–356. 10.1107/s2052520621002961 34096516

[B27] Haraguchi H. Ishikawa H. Mizutani K. Tamura Y. Kinoshita T. (1998). Antioxidative and superoxide scavenging activities of retrochalcones in *Glycyrrhiza inflata*. *Bioorg. Med. Chem.* 6 339–347. 10034-7 10.1016/s0968-0896 9568287

[B28] Hatano T. Shintani Y. Aga Y. Shiota S. Tsuchiya T. Yoshida T. (2000). Phenolic constituents of licorice. VIII. Structures of glicophenone and glicoisoflavanone, and effects of licorice phenolics on methicillin-resistant *Staphylococcus aureus*. *Chem. Pharm. Bull.* 48 1286–1292. 10.1248/cpb.48.1286 10993226

[B29] Haydakm M. H. (1936). Is wax a necessary constituent of the diet of wax moth larvae? *Ann. Entomol. Soc. Am.* 29 581–588. 10.1093/aesa/29.4.581 34249220

[B30] Heilmann C. Ziebuhr W. Becker K. (2019). Are coagulase-negative staphylococci virulent? *Clin. Microbiol. Infect.* 25 1071–1080. 10.1016/j.cmi.2018.11.012 30502487

[B31] Ignasiak K. Maxwell A. (2017). *Galleria mellonella* (greater wax moth) larvae as a model for antibiotic susceptibility testing and acute toxicity trials. *BMC Res. Notes* 10:428. 10.1186/s13104-017-2757-8 28851426 PMC5576310

[B32] Jin X. Zheng C. J. Song M. X. Wu Y. Sun L. P. Li Y. J. (2012). Synthesis and antimicrobial evaluation of L-phenylalanine-derived C5-substituted rhodanine and chalcone derivatives containing thiobarbituric acid or 2-thioxo-4-thiazolidinone. *Eur. J. Med. Chem.* 56 203–209. 22982124 10.1016/j.ejmech.2012.08.026

[B33] Junqueira J. C. (2012). *Galleria mellonella* as a model host for human pathogens. *Virulence* 3 474–476. 10.4161/viru.22493 23211681 PMC3524145

[B34] Kant R. Kumar D. Agarwal D. Gupta R. D. Tilak R. Awasthi S. K. (2016). Synthesis of newer 1,2,3-triazole linked chalcone and flavone hybrid compounds and evaluation of their antimicrobial and cytotoxic activities. *Eur. J. Med. Chem.* 113 34–49. 10.1016/j.ejmech.2016.02.041 26922227

[B35] Kaur D. C. Chate S. S. (2015). Study of antibiotic resistance pattern in methicillin resistant *Staphylococcus aureus* with special reference to newer antibiotic. *J. Glob. Infect. Dis.* 7 78–84. 10.4103/0974-777x.157245 26069428 PMC4448330

[B36] Khan S. N. Khan A. U. (2016). Breaking the spell: Combating multidrug resistant ‘superbugs’. *Front. Microbiol.* 7:174. 10.3389/fmicb.2016.00174 26925046 PMC4757689

[B37] Konieczny M. T. Konieczny W. Sabisz M. Skladanowski A. Wakiec R. Augustynowicz-Kopec E. (2007). Acid-catalyzed synthesis of oxathiolone fused chalcones. Comparison of their activity toward various microorganisms and human cancer cells line. *Eur. J. Med. Chem.* 42 729–733. 10.1016/j.ejmech.2006.12.014 17300856

[B38] Koudokpon H. Armstrong N. Dougnon T. V. Fah L. Hounsa E. Bankolé H. S. (2018). Antibacterial activity of chalcone and dihydrochalcone compounds from *Uvaria chamae* roots against multidrug-resistant bacteria. *Biomed Res. Int.* 2018:1453173. 10.1155/2018/1453173 30225246 PMC6129846

[B39] Krawczyk B. Wysocka M. Kotłowski R. Bronk M. Michalik M. Samet A. (2020). Linezolid-resistant *Enterococcus faecium* strains isolated from one hospital in Poland -commensals or hospital-adapted pathogens? *PLoS One* 15:e0233504. 10.1371/journal.pone.0233504 32453777 PMC7250452

[B40] Kucerova-Chlupacova M. Kunes J. Buchta V. Vejsova M. Opletalova V. (2015). Novel pyrazine analogs of chalcones: Synthesis and evaluation of their antifungal and antimycobacterial activity. *Molecules* 20 1104–1117. 10.3390/molecules20011104 25587786 PMC6272410

[B41] Kucerova-Chlupacova M. Vyskovska-Tyllova V. Richterova-Finkova L. Kunes J. Buchta V. Vejsova M. (2016). Novel halogenated pyrazine-based chalcones as potential antimicrobial drugs. *Molecules* 21:1421. 10.3390/molecules21111421 27801810 PMC6273737

[B42] Kumar S. Siji J. V. Nambisan B. Mohandas C. (2012). Activity and synergistic interactions of stilbenes and antibiotic combinations against bacteria *in vitro*. *World J. Microbiol. Biotechnol.* 28 3143–3150. 22806752 10.1007/s11274-012-1124-0

[B43] Lee G. S. Kim E. S. Cho S. I. Kim J. H. Choi G. Ju Y. S. (2010). Antibacterial and synergistic activity of prenylated chalcone isolated from the roots of *Sophora flavescens*. *J. Korean Soc. Appl. Biol. Chem.* 53 290–296. 10.3839/jksabc.2010.045

[B44] Liaras K. Geronikaki A. Glamoèlija J. Cirić A. Soković M. (2011). Thiazole-based chalcones as potent antimicrobial agents. Synthesis and biological evaluation. *Bioorg. Med. Chem.* 19 3135–3140. 10.1016/j.bmc.2011.04.007 21524583

[B45] Lipinski C. A. Lombardo F. Dominy B. W. Feeney P. J. (1997). Experimental and computational approaches to estimate solubility and permeability in drug discovery and development settings. *Adv. Drug Deliv. Rev.* 23 3–25. 00423-1 10.1016/S0169-409X 11259830

[B46] Liu X. F. Zheng C. J. Sun L. P. Liu X. K. Piao H. R. (2011). Synthesis of new chalcone derivatives bearing 2,4-thiazolidinedione and benzoic acid moieties as potential antibacterial agents. *Eur. J. Med. Chem.* 46 3469–3473. 10.1016/j.ejmech.2011.05.012 21624712

[B47] Löwdin E. Odenholt-Tornqvist I. Bengtsson S. Cars O. (1993). A new method to determine postantibiotic effect and effects of subinhibitory antibiotic concentrations. *Antimicrob. Agents Chemother.* 37 2200–2205. 10.1128/aac.37.10.2200 8257145 PMC192250

[B48] Mahapatra D. K. Bharti S. K. Asati V. (2017). Chalcone derivatives: Anti-inflammatory potential and molecular targets perspectives. *Curr. Top. Med. Chem.* 17 3146–3169. 10.2174/1568026617666170914160446 28914193

[B49] Matos M. J. Vazquez-Rodriguez S. Uriarte E. Santana L. (2015). Potential pharmacological uses of chalcones: A patent review (from June 2011-2014). *Expert Opin. Ther. Pat.* 25 351–366. 10.1517/13543776.2014.995627 25598152

[B50] Matuschek E. Brown D. F. Kahlmeter G. (2014). Development of the EUCAST disk diffusion antimicrobial susceptibility testing method and its implementation in routine microbiology laboratories. *Clin. Microbiol. Infect.* 20 O255–O266. 10.1111/1469-0691.12373 24131428

[B51] Mbaveng A. T. Ngameni B. Kuete V. Simo I. K. Ambassa P. Roy R. (2008). Antimicrobial activity of the crude extracts and five flavonoids from the twigs of *Dorstenia barteri* (Moraceae). *J. Ethnopharmacol.* 116 483–489. 10.1016/j.jep.2007.12.017 18280679

[B52] McGuinness W. A. Malachova N. DeLeo F. R. (2017). Vancomycin resistance in *Staphylococcus aureus*. *Yale J. Biol. Med.* 90 269–281. 28656013 PMC5482303

[B53] Meier D. Hernández M. V. van Geelen L. Muharini R. Proksch P. Bandow J. E. (2019). The plant-derived chalcone Xanthoangelol targets the membrane of Gram-positive bacteria. *Bioorg. Med. Chem.* 27:115151. 10.1016/j.bmc.2019.115151 31648878

[B54] Miller W. R. Munita J. M. Arias C. A. (2014). Mechanisms of antibiotic resistance in enterococci. *Expert Rev. Anti Infect. Ther.* 12 1221–1236. 10.1586/14787210.2014.956092 25199988 PMC4433168

[B55] Moawad A. A. Hotzel H. Awad O. Roesler U. Hafez H. M. Tomaso H. (2019). Evolution of antibiotic resistance of coagulase-negative staphylococci isolated from healthy turkeys in Egypt: First report of linezolid resistance. *Microorganisms* 7:476. 10.3390/microorganisms7100476 31652567 PMC6843140

[B56] Nielsen S. F. Boesen T. Larsen M. Schonning K. Kromann H. (2004). Antibacterial chalcones-bioisosteric replacement of the 4’-hydroxy group. *Bioorg. Med. Chem.* 12 3047–3054. 10.1016/j.bmc.2004.03.071 15142563

[B57] Nielsen S. F. Larsen M. Boesen T. Schonning K. Kromann H. (2005). Cationic chalcone antibiotics. Design, synthesis, and mechanism of action. *J. Med. Chem.* 48 2667–2677. 10.1021/jm049424k 15801857

[B58] Opletalova V. Hartl J. Patel A. Palat K. Buchta V. (2002). Ring substituted 3-phenyl-1-(2-pyrazinyl)-2-propen-1-ones as potential photosynthesis-inhibiting, antifungal and antimycobacterial agents. *Farmaco* 57 135–144. 01187-9 10.1016/s0014-827x 11902656

[B59] Opletalova V. Pour M. Kunes J. Buchta V. Silva L. Kralova K. (2006). Synthesis and biological evaluation of (E)-3-(nitrophenyl)-1-(pyrazin-2-yl)prop-2-en-1-ones. *Collect. Czechoslov. Chem. Commun.* 71 44–58.

[B60] Ouyang Y. Li J. Chen X. Fu X. Sun S. Wu Q. (2021). Chalcone derivatives: Role in anticancer therapy. *Biomolecules* 11:894. 10.3390/biom11060894 34208562 PMC8234180

[B61] Quiloan M. L. G. Vu J. Carvalho J. (2012). *Enterococcus faecalis* can be distinguished from *Enterococcus faecium* via differential susceptibility to antibiotics and growth and fermentation characteristics on mannitol salt agar. *Front. Biol.* 7 167–177. 10.1007/s11515-012-1183-5

[B62] Rukayadi Y. Lee K. Han S. Yong D. Hwang J. K. (2009). *In vitro* activities of panduratin a against clinical *Staphylococcus* strains. *Antimicrob. Agents Chemother.* 53 4529–4532. 10.1128/aac.00624-09 19651906 PMC2764148

[B63] Spangler S. K. Lin G. Jacobs M. R. Appelbaum P. C. (1998). Postantibiotic effect and postantibiotic sub-MIC effect of levofloxacin compared to those of ofloxacin, ciprofloxacin, erythromycin, azithromycin, and clarithromycin against 20 pneumococci. *Antimicrob. Agents Chemother.* 42 1253–1255. 10.1128/aac.42.5.1253 9593160 PMC105793

[B64] Stubbings W. J. Bostock J. M. Ingham E. Chopra I. J. (2004). Assessment of a microplate method for determining the post-antibiotic effect in *Staphylococcus aureus* and *Escherichia coli*. *Antimicrob. Chemother.* 54 139–143. 10.1093/jac/dkh275 15150167

[B65] Tsukiyama R. I. Katsura H. Tokuriki N. Kobayashi M. (2002). Antibacterial activity of licochalcone A against spore-forming bacteria. *Antimicrob. Agents Chemother.* 46 1226–1230. 10.1128/aac.46.5.1226-1230.2002 11959549 PMC127195

[B66] Ventola C. L. (2015). The antibiotic resistance crisis: Part 1: Causes and threats. *P T* 40 277–283. 25859123 PMC4378521

[B67] World Health Organization [WHO] (2021). *Antimicrobial resistance.* Available Online at: https://www.who.int/health-topics/antimicrobial-resistance [accessed April 2, 2022].

[B68] World Health Organization [WHO] (2017). *Prioritization of pathogens to guide discovery, research and development of new antibiotics for drug-resistant bacterial infections, including tuberculosis.* Available Online at: https://apps.who.int/iris/handle/10665/311820 [accessed April 2, 2022].

[B69] Xu M. Wu P. Shen F. Ji J. Rakesh K. P. (2019). Chalcone derivatives and their antibacterial activities: Current development. *Bioorg. Chem.* 91:103133. 10.1016/j.bioorg.2019.103133 31374524

[B70] Zhang M. M. Prior A. M. Maddox M. M. Shen W. J. Hevener K. E. Bruhn D. F. (2018). Pharmacophore modeling, synthesis, and antibacterial evaluation of chalcones and derivatives. *ACS Omega* 3 18343–18360. 10.1021/acsomega.8b03174 30613820 PMC6312637

[B71] Zhuang C. Zhang W. Sheng C. Zhang W. Xing C. Miao Z. (2017). Chalcone: A privileged structure in medicinal chemistry. *Chem. Rev.* 117 7762–7810. 10.1021/acs.chemrev.7b00020 28488435 PMC6131713

