# Supplementary material for: Comprehensive insight into anti-staphylococcal and anti-enterococcal action of brominated and chlorinated pyrazine-based chalcones
Source: Front Microbiol. 2022 Aug 17;13:912467. doi: 10.3389/fmicb.2022.912467 (PMC9428509; doi:10.3389/fmicb.2022.912467)
Supplement: Supplementary file 1 [file Data_Sheet_1.docx]

**Table S1.** List of reference strains and clinical isolates from the genus *Staphylococcus* and *Enterococcus* with their susceptibility/resistance profile used for CH-0y and CH-0w extended study of antibacterial action

| **ID No.** | **Bacterial strain** | **Susceptibility (S)/resistance (R) profile** | **Strain specification/clinical specimen** |
| --- | --- | --- | --- |
| ATCC 29213 | *Staphylococcus aureus* | *****S – nitrofurantoin, trimethoprim/sulfamethoxazole, tetracycline, gentamicin, clindamycin, erythromycin | reference laboratory strain/wound |
| ATCC 43300 | methicillin-resistant *Staphylococcus aureus* | *****S – nitrofurantoin, trimethoprim/sulfamethoxazole, tetracycline, linezolid  *****R – clindamycin, erythromycin | reference laboratory strain/NS |
| ATCC  29212 | *Enterococcus faecalis* | *****S – ampicillin, nitrofurantoin, vancomycin, linezolid, ciprofloxacin, tigecycline | reference laboratory strain/urine |
| CCM 1767 | vancomycin-resistant *Staphylococcus aureus* | S – ND  *****R – vancomycin, oxacillin | reference laboratory strain |
| 203/16 NIPH | vancomycin-resistant *Staphylococcus aureus* | S – ND  *****R – vancomycin, oxacillin | laryng swab |
| 131/16 | methicillin-resistant *Staphylococcus aureus* | ******S – nitrofurantoin, trimethoprim/sulfamethoxazole, tetracycline ******R – cefoxitin, clindamycin, erythromycin | throat swab |
| 136/16 | *Staphylococcus aureus* | ******S – cefoxitin, clindamycin, nitrofurantoin, trimethoprim/sulfamethoxazole, tetracycline ******R – erythromycin | laryng swab |
| 137/16 | *Staphylococcus aureus* | ******S – cefoxitin, clindamycin, erythromycin, nitrofurantoin, tetracycline, trimethoprim/sulfamethoxazole | burn wound |
| 138/16 | methicillin-resistant *Staphylococcus aureus* | ******S – clindamycin, nitrofurantoin, trimethoprim/sulfamethoxazole, tetracycline  ******R – cefoxitin, erythromycin | sputum |
| 196/16 | *Staphylococcus epidermidis* | ******S – tetracycline, nitrofurantoin  ******R – cefoxitin, erythromycin, clindamycin, trimethoprim/sulfamethoxazole | drain |
| 198/16 | vancomycin-resistant *Enterococcus faecium* | ******S – linezolid ******R – ampicillin, gentamicin, teicoplanin, tigecycline, vancomycin | catheter tube |
| 1/21 | *Staphylococcus lugdunensis* | ******S – oxacillin, tetracycline, trimethoprim/sulfamethoxazole, ceftaroline, gentamicin, ciprofloxacin, linezolid, rifampicin, tigecycline  ******R – erythromycin, clindamycin | central venous catheter |
| 3/21 | *Staphylococcus epidermidis* | ******S – tetracycline, ceftaroline, linezolid, rifampicin, tigecycline  ******R – oxacillin, erythromycin, clindamycin, trimethoprim/sulfamethoxazole, gentamicin, ciprofloxacin | blood |
| 4/21 | *Staphylococcus epidermidis* | ******S – erythromycin, clindamycin, tetracycline, ceftaroline, gentamicin, linezolid, rifampicin, tigecycline  ******R – oxacillin, trimethoprim/sulfamethoxazole, ciprofloxacin | femur |
| 5/21 | *Staphylococcus epidermidis* | ******S – oxacillin, erythromycin, clindamycin, tetracycline, trimethoprim/sulfamethoxazole, ceftaroline, gentamicin, ciprofloxacin, linezolid, rifampicin, tigecycline | blood |
| 17/21 | *Enterococcus faecium* | ******S – linezolid  ******R – ampicillin, nitrofurantoin, vancomycin, fosfomycin, teicoplanin | urine |
| 18/21 | *Enterococcus faecium* | ******S – daptomycin  ******R – vancomycin, linezolid, tigecycline, fosfomycin, ampicillin, nitrofurantoin, trimethoprim/sulfamethoxazole | urine |

ATCC No. – American Type Culture Collection number, ID No. – internal laboratory identification number, CCM – Czech Collection of Microorganisms, Czech Republic, NIPH – The National Institute of Public Health in Prague, Czech Republic, ND – not determined, NS – not specified, ***** evaluation of the susceptibility/resistance profile by microdilution broth method, according to EUCAST recommendation**,** susceptibility/resistance profile taken from German collection of microorganisms (DSM), and the associated database, *BacDive*, [BacDive | The Bacterial Diversity Metadatabase (dsmz.de)](https://bacdive.dsmz.de/), ****** disc diffusion test according to EUCAST recommendation

**Table S2****.** Total fractional inhibitory concentration index (FICI) determined by checkerboard assay of vancomycin (VAN) and CH-0y, trimethoprim/sulfamethoxazole (SXT) and CH-0y, ciprofloxacin (CIP) and CH-0y, rifampicin (RIF) and CH-0y, linezolid (LIN) and CH-0y combinations against methicillin-resistant *Staphylococcus aureus* (ATCC 43300). The minimum inhibitory concentration (MIC) of compounds alone corresponds to: MIC(VAN) = 1.346 μM, MIC(CH-0y) = 31.25 μM in VAN+CH-0y combination; MIC(SXT) = 3.680 μM and MIC(CH-0y) = 62.5μM in SXT+CH-0y combination; MIC(CIP) = 0.755 μM and MIC(CH-0y) = 31.25 μM in CIP+CH-0y combination and MIC(RIF) = 0.006 μM and MIC(CH-0y) = 62.5 μM in RIF+CH-0y combination, MIC(LIN) = 23.714 μM and MIC(CH-0y) = 31.25 μM in LIN+CH-0y combination.

|  |  | Combination of compounds (vancomycin : CH-0y) | | | | | |
| --- | --- | --- | --- | --- | --- | --- | --- |
| Concentration ratio  VAN : CH-0y | | | Concentration ratio  VAN : CH-0y | FIC(VAN) | FIC(CH-0y) | FICI | Effect |
| (μM) | | | (mg/L) |  |  |  |  |
| 0.005 : 31.25 | | | 0.008 : 9.035 | 0.004 | 1 | 1.004 | I |
| 0.011 : 31.25 | | | 0.016 : 9.035 | 0.008 | 1 | 1.008 | I |
| 0.021 : 31.25 | | | 0.031 : 9.035 | 0.016 | 1 | 1.016 | I |
| 0.0421 : 31.25 | | | 0.063 : 9.035 | 0.031 | 1 | 1.031 | I |
| 0.084 : 31.25 | | | 0.125 : 9.035 | 0.063 | 1 | 1.063 | I |
| 0.168 : 31.25 | | | 0.25 : 9.035 | 0.125 | 1 | 1.125 | I |
| 0.337 : 31.25 | | | 0.5 : 9.035 | 0.25 | 1 | 1.25 | I |
| 0.673 : 31.25 | | | 1 : 9.035 | 0.5 | 1 | 1.5 | I |
| 1.346 : 15.625 | | | 2 : 4.518 | 1 | 0.5 | 1.5 | I |
| 1.346 : 7.813 | | | 2 : 2.259 | 1 | 0.250 | 1.25 | I |
| 1.346 : 3.906 | | | 2 : 1.129 | 1 | 0.125 | 1.125 | I |
| 1.346 : 1.953 | | | 2 : 0.565 | 1 | 0.062 | 1.062 | I |
| 1.346 : 0.977 | | | 2 : 0.282 | 1 | 0.031 | 1.031 | I |
|  |  | Combination of compounds (trimethoprim/sulfamethoxazole : CH-0y) | | | | | |
| Concentration ratio  SXT : CH-0y | | | Concentration ratio  SXT : CH-0y | FIC(SXT) | FIC(CH-0y) | FICI | Effect |
| (μM) | | | (mg/L) |  |  |  |  |
| 0.057 : 31.25 | | | 0.032 : 9.035 | 0.016 | 0.5 | 0.516 | AD |
| 0.115 : 125 | | | 0.063 : 36.141 | 0.031 | 2 | 2.031 | I |
| 0.230 : 31.25 | | | 0.125 : 9.035 | 0.063 | 0.5 | 0.563 | AD |
| 0.460 : 31.25 | | | 0.25 : 9.035 | 0.125 | 0.5 | 0.625 | AD |
| 0.920 : 31.25 | | | 0.5 : 9.035 | 0.25 | 0.5 | 0.75 | AD |
| 1.840 : 62.5 | | | 1 : 18.071 | 0.5 | 1 | 1.5 | I |
| 3.680 : 125 | | | 2 : 36.141 | 1 | 2 | 3 | I |
| 7.358 : 125 | | | 4 : 36.141 | 2 | 2 | 4 | I |
| 14.717 : 62.5 | | | 8 : 18.071 | 4 | 1 | 5 | AN |
| 3.679 : 31.25 | | | 2 : 9.035 | 1 | 0.5 | 1.5 | I |
| 3.679 : 15.625 | | | 2 : 4.518 | 1 | 0.25 | 1.25 | I |
| 14.716 : 7.813 | | | 8 : 2.259 | 4 | 0.125 | 4.125 | AN |
| 7.358 : 3.906 | | | 4 : 1.129 | 2 | 0.062 | 2.062 | I |
| 3.679 : 1.953 | | | 2 : 0.565 | 1 | 0.031 | 1.031 | I |
| 14.717 : 0.977 | | | 8 : 0.282 | 4 | 0.015 | 4.015 | AN |
|  |  | Combination of compounds (ciprofloxacin : CH-0y) | | | | | |
| Concentration ratio  CIP : CH-0y | | | Concentration ratio  CIP : CH-0y | FIC(CIP) | FIC(CH-0y) | FICI | Effect |
| (μM) | | | (mg/L) |  |  |  |  |
| 0.006 : 31.25 | | | 0.002 : 9.035 | 0.008 | 1 | 1.008 | I |
| 0.012 : 31.25 | | | 0.004 : 9.035 | 0.016 | 1 | 1.016 | I |
| 0.024 : 31.25 | | | 0.008 : 9.035 | 0.031 | 1 | 1.031 | I |
| 0.047 : 31.25 | | | 0.016 : 9.035 | 0.062 | 1 | 1.062 | I |
| 0.095 : 31.25 | | | 0.032 : 9.035 | 0.126 | 1 | 1.126 | I |
| 0.189 : 31.25 | | | 0.063 : 9.035 | 0.25 | 1 | 1.25 | I |
| 0.377 : 31.25 | | | 0.125 : 9.035 | 0.5 | 1 | 1.5 | I |
| 0.755 : 15.625 | | | 0.25 : 4.518 | 1 | 0.5 | 1.5 | I |
| 1.509 : 7.813 | | | 0.5 : 2.259 | 2 | 0.25 | 2.25 | I |
| 1.509 : 3.906 | | | 0.5 : 1.129 | 2 | 0.125 | 2.125 | I |
| 1.509 : 1.953 | | | 0.5 : 0.565 | 2 | 0.062 | 2.062 | I |
| 1.509 : 0.977 | | | 0.5 : 0.282 | 2 | 0.031 | 2.031 | I |
|  |  | Combination of compounds (rifampicin : CH-0y) | | | | | |
| Concentration ratio  RIF×10^-3^ : CH-0y | | | Concentration ratio  RIF×10^-3^ : CH-0y | FIC(RIF) | FIC(CH-0y) | FICI | Effect |
| (μM) | | | (mg/L) |  |  |  |  |
| 0.095 : 62.5 | | | 0.078 : 18.071 | 0.016 | 1 | 1.016 | I |
| 0.19 : 62.5 | | | 0.156 : 18.071 | 0.031 | 1 | 1.031 | I |
| 0.38 : 62.5 | | | 0.313 : 18.071 | 0.063 | 1 | 1.063 | I |
| 0.759 : 62.5 | | | 0.625 : 18.071 | 0.125 | 1 | 1.125 | I |
| 1.519 : 62.5 | | | 1.25 : 18.071 | 0.25 | 1 | 1.25 | I |
| 3.038 : 31.25 | | | 2.5 : 9.035 | 0.5 | 0.5 | 1 | I |
| 6.076 : 15.625 | | | 5 : 4.518 | 1 | 0.25 | 1.25 | I |
| 24.303 : 15.625 | | | 20 : 4.518 | 4 | 0.25 | 4.25 | AN |
| 24.303 : 7.813 | | | 20 : 2.259 | 4 | 0.125 | 4.125 | AN |
| 12.151 : 3.906 | | | 10 : 1.129 | 2 | 0.063 | 2.063 | I |
| 12.151 : 1.953 | | | 10 : 0.565 | 2 | 0.031 | 2.031 | I |
| 12.151 : 0.977 | | | 10 : 0.282 | 2 | 0.016 | 2.016 | I |
|  |  | Combination of compounds (linezolid : CH-0y) | | | | | |
| Concentration ratio  LIN : CH-0y | | | Concentration ratio  LIN: CH-0y | FIC(LIN) | FIC(CH-0y) | FICI | Effect |
| (μM) | | | (mg/L) |  |  |  |  |
| 0.185 : 31.25 | | | 0.063 : 9.035 | 0.008 | 1 | 1.008 | I |
| 0.371 : 31.25 | | | 0.125 : 9.035 | 0.016 | 1 | 1.016 | I |
| 0.741 : 31.25 | | | 0.25 : 9.035 | 0.031 | 1 | 1.031 | I |
| 1.482 : 31.25 | | | 0.5 : 9.035 | 0.063 | 1 | 1.063 | I |
| 2.964 : 31.25 | | | 1 : 9.035 | 0.125 | 1 | 1.125 | I |
| 5.929 : 31.25 | | | 2 : 9.035 | 0.25 | 1 | 1.25 | I |
| 11.857 : 31.25 | | | 4 : 9.035 | 0.5 | 1 | 1.5 | I |
| 23.714 : 15.625 | | | 8 : 4.518 | 1 | 0.5 | 1.5 | I |
| 23.714 : 7.813 | | | 8 : 2.259 | 1 | 0.25 | 1.25 | I |
| 23.714 : 3.906 | | | 8 : 1.129 | 1 | 0.125 | 1.125 | I |
| 23.714 : 1.953 | | | 8 : 0.565 | 1 | 0.063 | 1.063 | I |
| 23.714 : 0.977 | | | 8 : 0.282 | 1 | 0.031 | 1.031 | I |

I – indifferent effect, AD – additive effect, AN – antagonistic effect, FIC – fractional inhibitory concentration, FICI – fractional inhibitory concentration index, FIC(VAN/SXT/CIP/RIF/LIN/CH-0y) = MIC of the combination / MIC(VAN/SXT/CIP/RIF/LIN/CH-0y) alone, FICI = FIC(VAN/SXT/CIP/RIF/LIN) + FIC(CH-0y). The effect was interpreted as follows: synergy when FICI ≤ 0.5, an additive effect when 0.5 > FICI ≤ 1, an indifferent effect when 1 < FICI ≤4, an antagonistic effect when FICI > 4

**Table S3.** Total fractional inhibitory concentration index (FICI) determined by checkerboard assay of vancomycin (VAN) and CH-0w, trimethoprim/sulfamethoxazole (SXT) and CH-0w, ciprofloxacin (CIP) and CH-0w, rifampicin (RIF) and CH-0w, linezolid (LIN) and CH-0w combinations against methicillin-resistant *Staphylococcus aureus* (ATCC 43300). The minimum inhibitory concentration (MIC) of compounds alone corresponds to: MIC(VAN) = 0.69 μM, MIC(CH-0w) = 31.25 μM in VAN+CH-0w combination; MIC(SXT) = 3.679 μM and MIC(CH-0w) = 31.25 μM in SXT+CH-0w combination; MIC(CIP) = 1.509 μM and MIC(CH-0w) = 31.25 μM in CIP+CH-0w combination and MIC(RIF) = 0.006 μM and MIC(CH-0w) = 31.25 μM in RIF+CH-0w combination, MIC(LIN) = 23.714 μM and MIC(CH-0w) = 31.25 μM in LIN+CH-0w combination

|  |  | Combination of compounds (vancomycin : CH-0w) | | | | | |
| --- | --- | --- | --- | --- | --- | --- | --- |
| Concentration ratio  VAN : CH-0w | | | Concentration ratio  VAN : CH-0w | FIC(VAN) | FIC(CH-0w) | FICI | Effect |
| (μM) | | | (mg/L) |  |  |  |  |
| 0.005 : 62.5 | | | 0.008 : 15.293 | 0.008 | 2 | 2.008 | I |
| 0.011 : 62.5 | | | 0.016 : 15.293 | 0.016 | 2 | 2.016 | I |
| 0.022 : 62.5 | | | 0.032 : 15.293 | 0.032 | 2 | 2.032 | I |
| 0.043 : 62.5 | | | 0.063 : 15.293 | 0.063 | 2 | 2.063 | I |
| 0.086 : 62.5 | | | 0.125 : 15.293 | 0.125 | 2 | 2.125 | I |
| 0.173 : 62.5 | | | 0.25 : 15.293 | 0.25 | 2 | 2.25 | I |
| 0.345 : 62.5 | | | 0.5 : 15.293 | 0.5 | 2 | 2.5 | I |
| 0.69 : 31.25 | | | 1 : 7.646 | 1 | 1 | 2 | I |
| 1.38 : 15.625 | | | 2 : 3.823 | 2 | 0.5 | 2.5 | I |
| 1.38 : 7.813 | | | 2 : 1.912 | 2 | 0.25 | 2.25 | I |
| 0.69 : 3.906 | | | 1 : 0.956 | 1 | 0.125 | 1.125 | I |
| 0.69 : 1.953 | | | 1 : 0.478 | 1 | 0.062 | 1.062 | I |
| 1.38 : 0.977 | | | 2 : 0.239 | 2 | 0.031 | 2.031 | I |
|  |  | Combination of compounds (trimethoprim/sulfamethoxazole : CH-0w) | | | | | |
| MIC (SXT : CH-0w) | | | MIC (SXT : CH-0w) | FIC(SXT) | FIC(CH-0w) | FICI | Effect |
| μM | | | mg/L |  |  |  |  |
| 0.058 : 62.5 | | | 0.032 : 15.293 | 0.016 | 2 | 2.016 | I |
| 0.115 : 31.25 | | | 0.063 : 7.646 | 0.031 | 1 | 1.031 | I |
| 0.230 : 62.5 | | | 0.125 : 15.293 | 0.063 | 2 | 2.063 | I |
| 0.46 : 62.5 | | | 0.25 : 15.293 | 0.125 | 2 | 2.125 | I |
| 0.92 : 125 | | | 0.5 : 30.585 | 0.25 | 4 | 4.25 | AN |
| 1.84 : 62.5 | | | 1 : 15.293 | 0.5 | 2 | 2.5 | I |
| 3.68 : 31.25 | | | 2 : 7.646 | 1 | 1 | 2 | I |
| 3.68 : 15.625 | | | 2 : 3.823 | 1 | 0.5 | 1.5 | I |
| 3.68 : 7.813 | | | 2 : 1.912 | 1 | 0.25 | 1.25 | I |
| 3.68 : 3.906 | | | 2 : 0.956 | 1 | 0.125 | 1.125 | I |
| 3.68 : 1.953 | | | 2 : 0.478 | 1 | 0.063 | 1.063 | I |
| 3.68 : 0.977 | | | 2 : 0.239 | 1 | 0.031 | 1.031 | I |
|  |  | Combination of compounds (ciprofloxacin : CH-0w) | | | | | |
| MIC (CIP : CH-0w) | | | MIC (CIP : CH-0w) | FIC(CIP) | FIC(CH-0w) | FICI | Effect |
| μM | | | mg/L |  |  |  |  |
| 0.006 : 31.25 | | | 0.002 : 7.646 | 0.004 | 1 | 1.004 | I |
| 0.012 : 62.5 | | | 0.004 : 15.293 | 0.008 | 2 | 2.008 | I |
| 0.024 : 62.5 | | | 0.008 : 15.293 | 0.016 | 2 | 2.016 | I |
| 0.047 : 62.5 | | | 0.016 : 15.293 | 0.031 | 2 | 2.031 | I |
| 0.095 : 62.5 | | | 0.032 : 15.293 | 0.063 | 2 | 2.063 | I |
| 0.189 : 62.5 | | | 0.063 : 15.293 | 0.125 | 2 | 2.125 | I |
| 0.377 : 31.25 | | | 0.125 : 7.646 | 0.25 | 1 | 1.25 | I |
| 1.509 : 31.25 | | | 0.5 : 7.646 | 1 | 1 | 2 | I |
| 1.509 : 15.625 | | | 0.5 : 3.823 | 1 | 0.5 | 1.5 | I |
| 1.509 : 7.81 | | | 0.5 : 1.911 | 1 | 0.25 | 1.25 | I |
| 1.509 : 3.906 | | | 0.5 : 0.478 | 1 | 0.062 | 1.062 | I |
| 0.755 : 1.953 | | | 0.25 : 0.239 | 0.5 | 0.031 | 0.531 | I |
| 1.509 : 0.977 | | | 0.5 : 0.239 | 1 | 0.031 | 1.031 | I |
|  |  | Combination of compounds (rifampicin : CH-0w) | | | | | |
| MIC (RIF×10^-3^ : CH-0w) | | | MIC (RIF×10^-3^ : CH-0w) | FIC(RIF) | FIC(CH-0w) | FICI | Effect |
| μM | | | mg/L |  |  |  |  |
| 0.095 : 31.25 | | | 0.078 : 7.646 | 0.016 | 1 | 1.016 | I |
| 0.190 : 62.5 | | | 0.156 : 15.293 | 0.031 | 2 | 2.031 | I |
| 0.380 : 62.5 | | | 0.313 : 15.293 | 0.063 | 2 | 2.063 | I |
| 0.76 : 62.5 | | | 0.625 : 15.293 | 0.125 | 2 | 2.125 | I |
| 1.52 : 62.5 | | | 1.25 : 15.293 | 0.25 | 2 | 2.25 | I |
| 3.038 : 31.25 | | | 2.5 : 7.646 | 0.5 | 1 | 1.5 | I |
| 6.076 : 15.625 | | | 5 : 3.823 | 1 | 0.5 | 1.5 | I |
| 6.076 : 7.813 | | | 5 : 1.912 | 1 | 0.25 | 1.250 | I |
| 6.076 : 3.906 | | | 5 : 0.956 | 1 | 0.125 | 1.125 | I |
| 6.076 : 1.953 | | | 5 : 0.478 | 1 | 0.062 | 1.062 | I |
| 6.076 : 0.977 | | | 5 : 0.239 | 1 | 0.031 | 1.031 | I |
|  |  | Combination of compounds (linezolid : CH-0w) | | | | | |
| MIC (LIN : CH-0w) | | | MIC (LIN: CH-0w) | FIC(LIN) | FIC(CH-0w) | FICI | Effect |
| μM | | | mg/L |  |  |  |  |
| 0.185 : 31.25 | | | 0.063 : 7.646 | 0.008 | 1 | 1.008 | I |
| 0.37 : 31.25 | | | 0.125 : 7.646 | 0.016 | 1 | 1.016 | I |
| 0.741 : 31.25 | | | 0.25 : 7.646 | 0.031 | 1 | 1.031 | I |
| 1.482 : 31.25 | | | 0.5 : 7.646 | 0.063 | 1 | 1.063 | I |
| 2.964 : 31.25 | | | 1 : 7.646 | 0.125 | 1 | 1.125 | I |
| 5.929 : 31.25 | | | 2 : 7.646 | 0.25 | 1 | 1.25 | I |
| 11.857 : 31.25 | | | 4 : 7.646 | 0.5 | 1 | 1.5 | I |
| 23.714 : 15.625 | | | 8 : 3.823 | 1 | 0.5 | 1.5 | I |
| 23.714 : 7.813 | | | 8 : 1.912 | 1 | 0.25 | 1.25 | I |
| 23.714 : 3.906 | | | 8 : 0.956 | 1 | 0.125 | 1.125 | I |
| 23.714 : 1.953 | | | 8 : 0.478 | 1 | 0.062 | 1.062 | I |
| 23.714 : 0.977 | | | 8 : 0.239 | 1 | 0.031 | 1.031 | I |

I – indifferent effect, AN – antagonistic effect, FIC – fractional inhibitory concentration, FICI – fractional inhibitory concentration index, FIC(VAN/SXT/CIP/RIF/LIN/CH-0y) = MIC of the combination / MIC(VAN/SXT/CIP/RIF/LIN/CH-0y) alone, FICI = FIC(VAN/SXT/CIP/RIF/LIN) + FIC(CH-0y). The effect was interpreted as follows: synergy when FICI ≤ 0.5, an additive effect when 0.5 > FICI ≤ 1, an indifferent effect when 1 < FICI ≤4, an antagonistic effect when FICI > 4

**Table S4.** Evaluation of *in vivo* toxicity of CH-0y **(A)** and CH-0w **(B)** using an animal model, larvae of *Galleria mellonella*. The tested compounds were administered *via* the intra-hemocoel route, through the last left proleg. After administration, larvae were incubated at 37 °C for five days and inspected after 24, 48, and 120 hours of incubation.

| **(A)** |  |  |  |  |  |  |  |  |
| --- | --- | --- | --- | --- | --- | --- | --- | --- |
| Mean dose of CH-0y  (mg/kg of body weight) | 1460.6 | 355.75 | 303 | 97.82 | 32.948 | 3.1 | Control group- 10 μl of PBS + 30% (v/v) DMSO | Control group- w/o any administration |
| Mortality (%) | (%) | (%) | (%) | (%) | (%) | (%) | (%) | (%) |
| Hours (visual inspection after administration) |  |  |  |  |  |  |  |  |
| 24 | 25 | 0 | 0 | 0 | 0 | 0 | 0 | 0 |
| 48 | 25 | 0 | 14.286 | 14.286 | 0 | 0 | 0 | 0 |
| 120 | 25 | 14.286 | 28.571 | 14.286 | 0 | 0 | 0 | 0 |
| **(B)** |  |  |  |  |  |  |  |  |
| Mean dose of CH-0w  (mg/kg of body weight) | 1813.1 | 379.8 | 240.02 | 118.4 | 37.5 | 3.65 | Control group- 10 μl of PBS + 30% (v/v) DMSO | Control group- w/o any administration |
| Mortality (%) | (%) | (%) | (%) | (%) | (%) | (%) | (%) | (%) |
| Hours (visual inspection after administration) |  |  |  |  |  |  |  |  |
| 24 | 20 | 14.286 | 0 | 14.286 | 0 | 0 | 0 | 0 |
| 48 | 40 | 14.286 | 0 | 14.286 | 0 | 0 | 0 | 0 |
| 120 | 40 | 28.571 | 14.286 | 28.571 | 33.333 | 28.571 | 0 | 0 |
|  |  |  |  |  |  |  |  |  |

**Table S5:** Survival analyses of *Galleria mellonella* larvae after administration of CH-0y. Values represent outputs from pairwise comparison Log-rank Mantel-Cox test and Hazard Ratio(s) (Mantel-Haenszel) test, with CI 95%. **ns** – no significant difference

| **CH-0y (mg/kg of body weight)** | | | | | | |
| --- | --- | --- | --- | --- | --- | --- |
|  | **3.1 (B)** | **32.948 (C)** | **97.82 (D)** | **303 (E)** | **355.75 (F)** | **1460.6 (G)** |
| **0**  **(control group – 30% DMSO + PBS (A))** | **ns**, χ^2^ (1) = 0.000  P > 0.9999  A/B = undefined  B/A = undefined | **ns**, χ^2^ (1) = 0.000  P > 0.9999  A/C = undefined  C/A = undefined | **ns**, χ^2^ (1) = 1.000  P = 0.3173  A/D = 0.1353  (0.002685–6.821)  D/A = 7.389  (0.1466–372.4) | **ns**, χ^2^ (1) = 2.163  P = 0.1413  A/E = 0.1245  (0.007758–1.999)  E/A = 8.029  (0.5001–128.9) | **ns**, χ^2^ (1) = 1.000  P = 0.3173  A/F = 0.1353  (0.002685–6.821)  F/A = 7.389  (0.1466–372.4) | **ns**, χ^2^ (1) = 1.750  P = 0.1859  A/G = 0.06393  (0.001087–3.760)  G/A = 15.64 (0.2659–920.1) |
| **3.1 (B)** |  | **ns**, χ^2^ (1) = 0.000  P > 0.9999  B/C = undefined  C/B = undefined | **ns**, χ^2^ (1) = 1.000  P = 0.3173  B/D = 0.1353  (0.002685–6.821)  D/B = 7.389  (0.1466–372.4) | **ns**, χ^2^ (1) = 2.163  P = 0.1413  B/E = 0.1245  (0.007758–1.999)  E/B = 8.029  (0.5001–128.9) | **ns**, χ^2^ (1) = 1.000  P = 0.3173  B/F = 0.1353  (0.002685–6.821)  F/B = 7.389  (0.1466–372.4) | **ns**, χ^2^ (1) = 1.750  P = 0.1859  B/G = 0.06393  (0.001087–3.760)  G/B = 15.64  (0.2659–920.1) |
| **32.948 (C)** |  |  | **ns**, χ^2^ (1) = 1.000  P = 0.3173  C/D = 0.1353  (0.002685–6.821)  D/C = 7.389  (0.1466–372.4) | **ns**, χ^2^ (1) = 2.163  P = 0.1413  C/E = 0.1245  (0.007758–1.999)  E/C = 8.029  (0.5001–128.9) | **ns**, χ^2^ (1) = 1.000  P = 0.3173  C/F = 0.1353  (0.002685–6.821)  F/C = 7.389  (0.1466–372.4) | **ns**, χ^2^ (1) = 1.750  P = 0.1859  C/G = 0.06393  (0.001087–3.760)  G/C = 15.64  (0.2659–920.1) |
| **97.82 (D)** |  |  |  | **ns**, χ^2^ (1) = 0.351  P = 0.5533  D/E = 0.4952  (0.04850–5.058)  E/D = 2.019  (0.1977–20.62) | **ns**, χ^2^ (1) = 0.003  P = 0.9566  D/F = 1.080  (0.06729–17.34)  F/D = 0.9257  (0.05766–14.86) | **ns**, χ^2^ (1) = 0.256  P = 0.6127  D/G = 0.4667  (0.02443–8.918)  G/D = 2.143  (0.1121–40.94) |
| **303 (E)** |  |  |  |  | **ns**, χ^2^ (1) = 0.472  P = 0.4922  E/F = 2.265  (0.2197–23.36)  F/E = 0.4415  (0.04281–4.553) | **ns**, χ^2^ (1) = 0.000  P = 0.9970  E/G = 0.9954  (0.08977–11.04)  G/E = 1.005  (0.09059–11.14) |
| **355.75 (F)** |  |  |  |  |  | **ns**, χ^2^ (1) = 0.256  P = 0.6127  F/G = 0.4667  (0.02443–8.918)  G/F = 2.143  (0.1121–40.94) |
| **1460.6 (G)** |  |  |  |  |  |  |

**Table S6**: Survival analyses of *Galleria mellonella* larvae after administration of CH-0w. Values represent outputs from pairwise comparison Log-rank Mantel-Cox test and Hazard Ratio(s) (Mantel-Haenszel) test, with CI 95%. **ns** – no significant difference

| **CH-0w (mg/kg of body weight)** | | | | | | |
| --- | --- | --- | --- | --- | --- | --- |
|  | **3.65 (B)** | **37.5 (C)** | **118.4 (D)** | **240.02 (E)** | **379.8 (F)** | **1813.1 (G)** |
| **0**  **(control group – 30% DMSO + PBS (A))** | **ns**, χ^2^ (1) = 2.167  P = 0.1410  A/B = 0.1146  (0.006398–2.051)  B/A = 8.729  (0.4875–156.3) | **ns**, χ^2^ (1) = 2.545  P = 0.1106  A/C = 0.09408  (0.005157–1.716)  C/A = 10.63  (0.5827–193.9) | **ns**, χ^2^ (1) = 2.163  P = 0.1413  A/D = 0.1245  (0.007758–1.99)  D/A = 8.029  (0.5001–128.9) | **ns**, χ^2^ (1) = 1.000  P = 0.3173  A/E = 0.1353  (0.002685–6.821)  E/A = 7.389  (0.1466–372.4) | **ns**, χ^2^ (1) = 2.154  P = 0.1422  A/F = 0.1245  (0.007758–1.999)  F/A = 8.029  (0.5001–128.9) | **ns**, χ^2^ (1) = 3.135  P = 0.0766  A/G = 0.07648  (0.004444–1.316)  G/A = 13.08  (0.7597–225.0) |
| **3.65 (B)** |  | **ns**, χ^2^ (1) = 0.03175  P = 0.8586  B/C = 0.8135  (0.08405–7,874)  C/B = 1.229  (0.1270–11.90) | **ns**, χ^2^ (1) = 0.0153  P = 0.9016  B/D = 0.8760  (0.1073–7.152)  D/B = 1.142  (0.1398–9.320) | **ns**, χ^2^ (1) = 0.394  P = 0.5302  B/E = 2.199  (0.1878–25.75)  E/B = 0.4548  (0.03884–5.325) | **ns**, χ^2^ (1) = 0.015  P = 0.9016  B/F = 0.8760  (0.1073–7.152)  F/B = 1.142  (0.1398–9.320) | **ns**, χ^2^ (1) = 0.453  P = 0.5009  B/G = 0.4815  (0.05729–4.046)  G/B = 2.077  (0.2472–17.46) |
| **37.5 (C)** |  |  | **ns**, χ^2^ (1) = 0.002  P = 0.9670  C/D = 1.046  (0.1267–8.632)  D/C = 0.9564  (0.1158–7.895) | **ns**, χ^2^ (1) = 0.609  P = 0.4350  C/E = 2.693  (0.2240–32.36)  E/C = 0.3714  (0.03090–4.464) | **ns**, χ^2^ (1) = 0.002  P = 0.9670  C/F = 1.046  (0.1267–8,632)  F/C = 0.9564  (0.1158–7.895) | **ns**, χ^2^ (1) = 0.261  P = 0.6091  C/G = 0.5792  (0.07142–4.698)  G/C = 1.726  (0.2129–14.00) |
| **118.4 (D)** |  |  |  | **ns**, χ^2^ (1) = 0.4717  P = 0.4922  D/E = 2.265  (0.2197–23.36)  E/D = 0.4415  (0.04281–4.553) | **ns**, χ^2^ (1) = 0.000  P > 0.9999  D/F = 1.000  (0.1290–7.751)  F/D = 1.000  (0.1290–7.751) | **ns**, χ^2^ (1) = 0.208  P = 0.6486  D/G = 0.6192  (0.07882–4.865)  G/D = 1.615  (0.2056–12.69) |
| **240.02 (E)** |  |  |  |  | **ns**, χ^2^ (1) = 0.472  P = 0.4922  E/F = 0.4415  (0.04281–4.553)  F/E = 2.265  (0.2197–23.36) | **ns**, χ^2^ (1) = 1.236  P = 0.2663  E/G = 0.2609  (0.02441–2.788)  G/E = 3.833  (0.3586–40.97) |
| **379.8 (F)** |  |  |  |  |  | **ns**, χ^2^ (1) = 0.208  P = 0.6486  F/G = 0.6192  (0.07882–4.865)  G/F = 1.615  (0.2056–12.69) |
| **1813.1 (G)** |  |  |  |  |  |  |


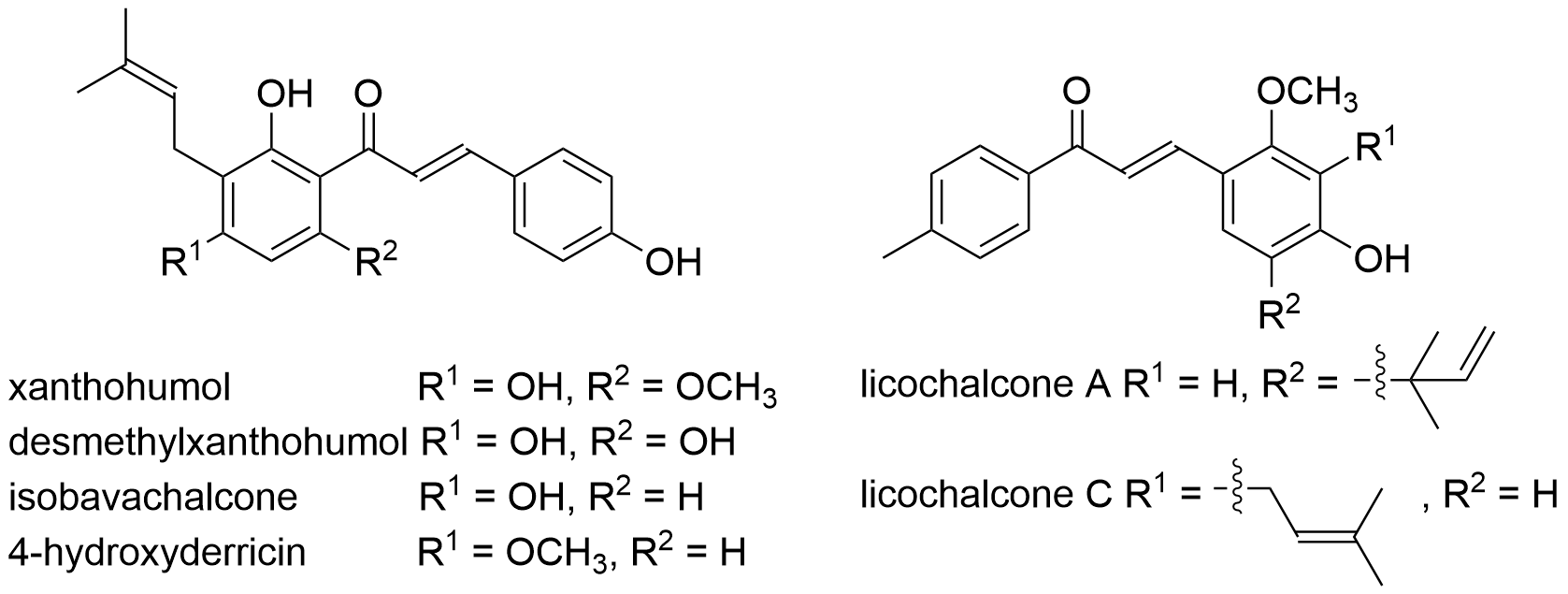


**Figure S1.** Natural chalcones with inhibitory activity against *Staphylococcus aureus*

**Figure S2**: Impact of different concentrations of CH-0y on the growth of selected staphylococcal and enterococcal bacterial strains. The interactions were evaluated after 20 hours of cultivation *in vitro*, at static incubation in a dark and humidified atmosphere at 36.8 °C. The values of initial optical density (O.D.) registered at the beginning of incubation were subtracted from the O.D. values obtained after 20 hours of cultivation. VRSA – vancomycin-resistant *Staphylococcus aureus*, VRE – vancomycin-resistant *Enterococcus* sp., MRSA – methicillin-resistant *Staphylococcus aureus*

**Figure S3**: Impact of different concentrations of CH-0w on the growth of selected staphylococcal and enterococcal bacterial strains. The interactions were evaluated after 20 hours of cultivation *in vitro*, at static incubation in a dark and humidified atmosphere at 36.8 °C. The values of initial optical density (O.D.) registered at the beginning of incubation were subtracted from the O.D. values obtained after 20 hours of cultivation. VRSA – vancomycin-resistant *Staphylococcus aureus*, VRE – vancomycin-resistant *Enterococcus* sp., MRSA – methicillin-resistant *Staphylococcus aureus*

**(A)**

**(B)**

**(C)**

**(D)**

**(E)**

**Figure S4.** Two-color heat maps of checkerboard MIC assays. Heat plot of two compound interactions, CH-0y with **(A)** vancomycin (VAN), **(B)** ciprofloxacin (CIP), **(C)** linezolid (LIN), **(D)** trimethoprim/sulfamethoxazole (SXT), and **(E)** rifampicin (RIF) with bacterial strain, methicillin-resistant *Staphylococcus aureus* (ATCC 43300), is demonstrated as percentage inhibition of the growth with comparison to a positive control (the growth of bacteria not limited by the antibacterial action of tested compounds) and evaluated after 20 hours of incubation. The minimum inhibitory concentration (MIC) of compounds alone corresponded to: VAN+CH-0y, MIC(VAN) = 1.346 μM, and MIC(CH-0y) = 31.25 μM; CIP+CH-0y, MIC(CIP) = 0.755 μM, and MIC(CH-0y) = 31.25 μM; LIN+CH-0y, MIC(LIN) = 23.714 μM, and MIC(CH-0y) = 31.25 μM; SXT+CH-0y, MIC(SXT) = 3.680 μM, and MIC(CH-0y) = 62.5 μM; RIF+CH-0y, MIC(RIF) = 0.006 μM, and MIC(CH-0y) = 62.5 μM.

**(A)**

**(B)**

** (C)**

**(D)**

**(E)**

**Figure S5.** Two-color heat maps of checkerboard MIC assays. Heat plot of two compound interactions, CH-0w with **(A)** vancomycin (VAN), **(B)** ciprofloxacin (CIP), **(C)** linezolid (LIN), **(D)** trimethoprim/sulfamethoxazole (SXT), and **(E)** rifampicin (RIF) with bacterial strain, methicillin-resistant *Staphylococcus aureus* (ATCC 43300), is demonstrated as percentage inhibition of the growth with comparison to a positive control (the growth of bacteria not limited by the antibacterial action of tested compounds) and evaluated after 20 hours of incubation. The minimum inhibitory concentration (MIC) of compounds alone corresponded to: VAN+CH-0w, MIC(VAN) = 0.69 μM, and MIC(CH-0w) = 31.25 μM; CIP+CH-0w, MIC(CIP) = 1.509 μM, and MIC(CH-0w) = 31.25 μM; LIN+CH-0w, MIC(LIN) = 23.714 μM, and MIC(CH-0w) = 31.25 μM; SXT+CH-0w, MIC(SXT) = 3.679 μM, and MIC(CH-0w) = 31.25 μM; RIF+CH-0w, MIC(RIF) = 0.006 μM, and MIC(CH-0w) = 31.25 μM.


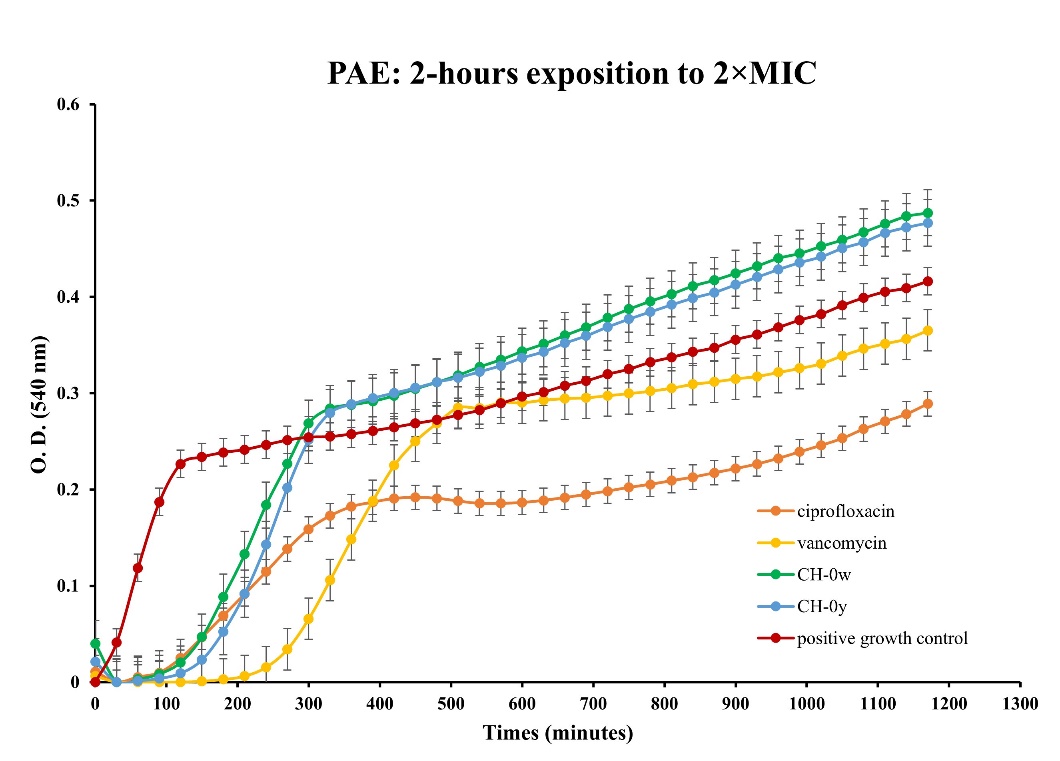

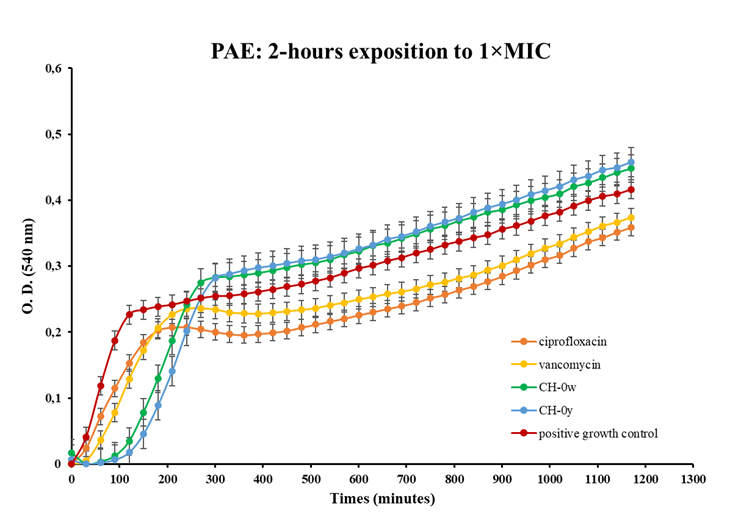


**(B)**

**(A)**

**Figure S****6.** Mapping of the growth of bacterial strain, *Staphylococcus aureus* (MRSA) ATCC 43300 after 2 hours of exposure at 2×MIC **(A)**, and 1×MIC **(B)** of CH-0y, CH-0w, and conventional drugs, vancomycin, and ciprofloxacin. The growth of bacterial biomass was spectrophotometrically detected at wavelength 540 nm. The number of replicates, *n* = 6.


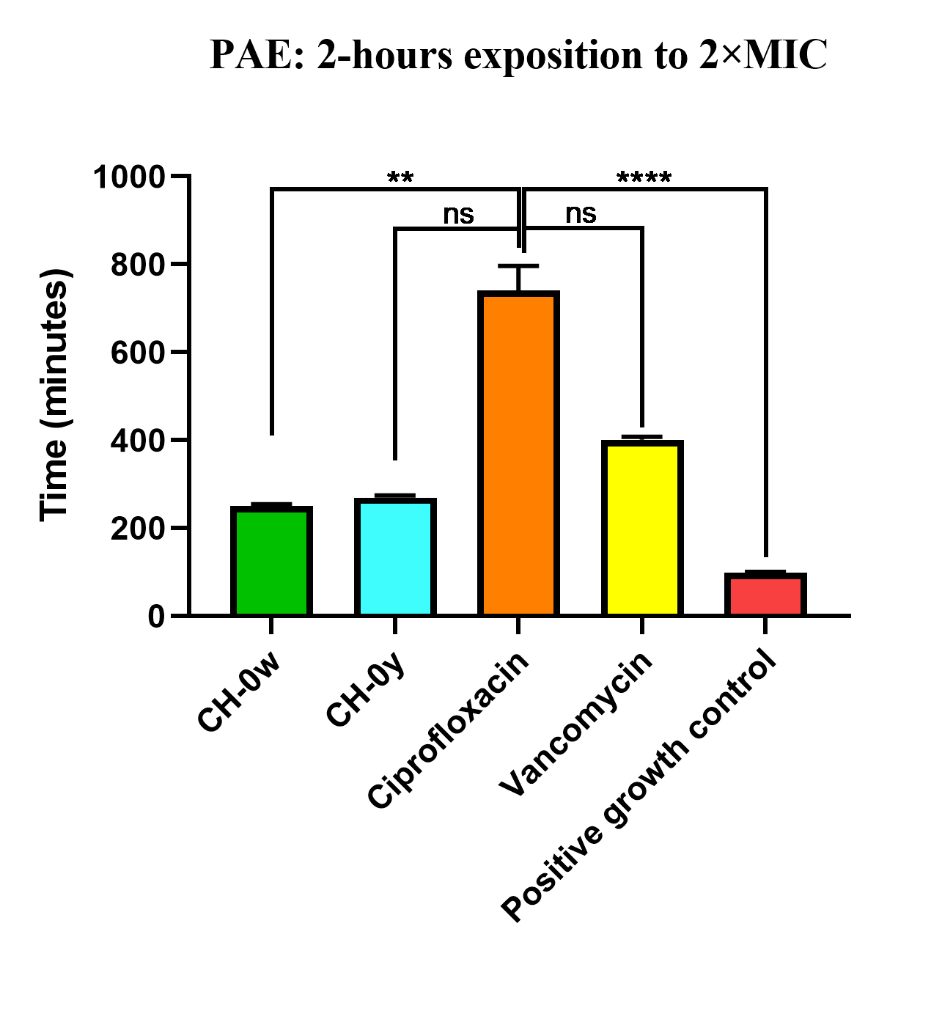


**Figure S7.** Comparison of the time growth delay after 2 hours exposure of methicillin-resistant *Staphylococcus aureus* (ATCC 43300) to the 2×MIC concentration of tested compounds, CH-0y and CH-0w. The antibiotic drug, ciprofloxacin, with a final concentration of 2×MIC, was employed as the reference compound. Vancomycin (final concentration 2×MIC), and positive growth control (unexposed bacteria) were also included in the study. One-way analysis of variance (ANOVA) with direct group-group comparison was employed for recognition of statistical significance (p-value <0.05). Error bars show SD. The number of replicates, *n* = 6.


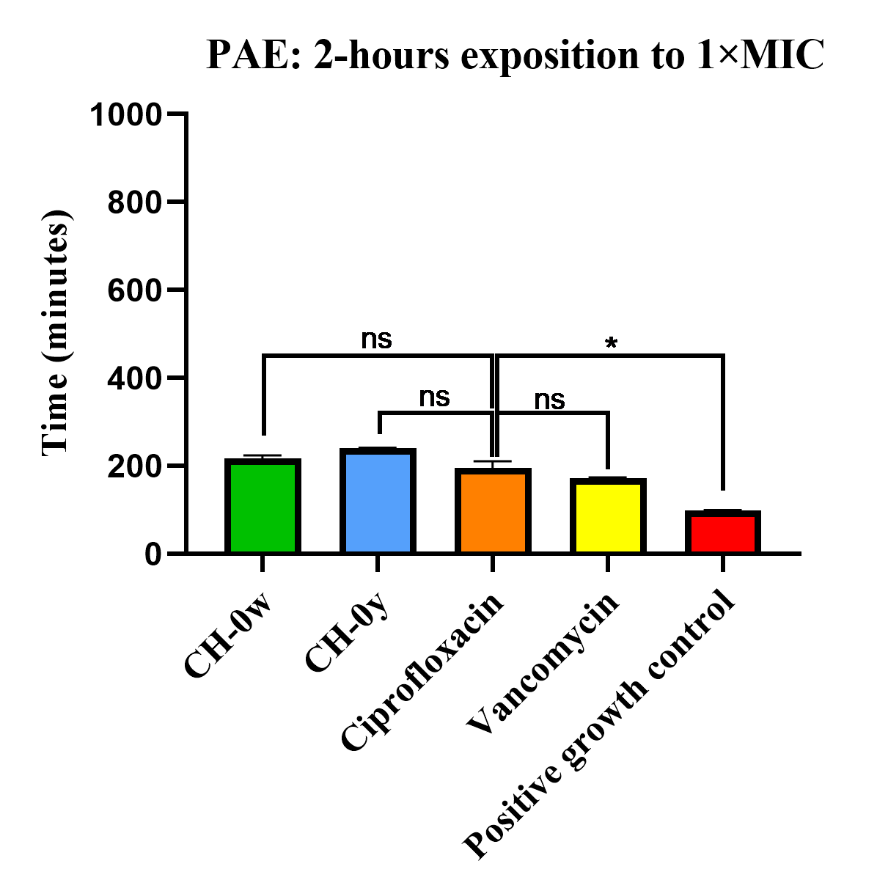


**Figure S8.** Comparison of the time growth delay after 2 hours exposure of methicillin-resistant *Staphylococcus aureus* (ATCC 43300) to the 1×MIC concentration of tested compounds, CH-0y and CH-0w. The antibiotic drug, ciprofloxacin, with a final concentration of 1×MIC, was employed as the reference compound. Vancomycin (final concentration 1×MIC), and positive growth control (unexposed bacteria) were also included in the study. One-way analysis of variance (ANOVA) with direct group-group comparison was employed for recognition of statistical significance (p-value <0.05). Error bars show SD. The number of replicates, *n* = 6.


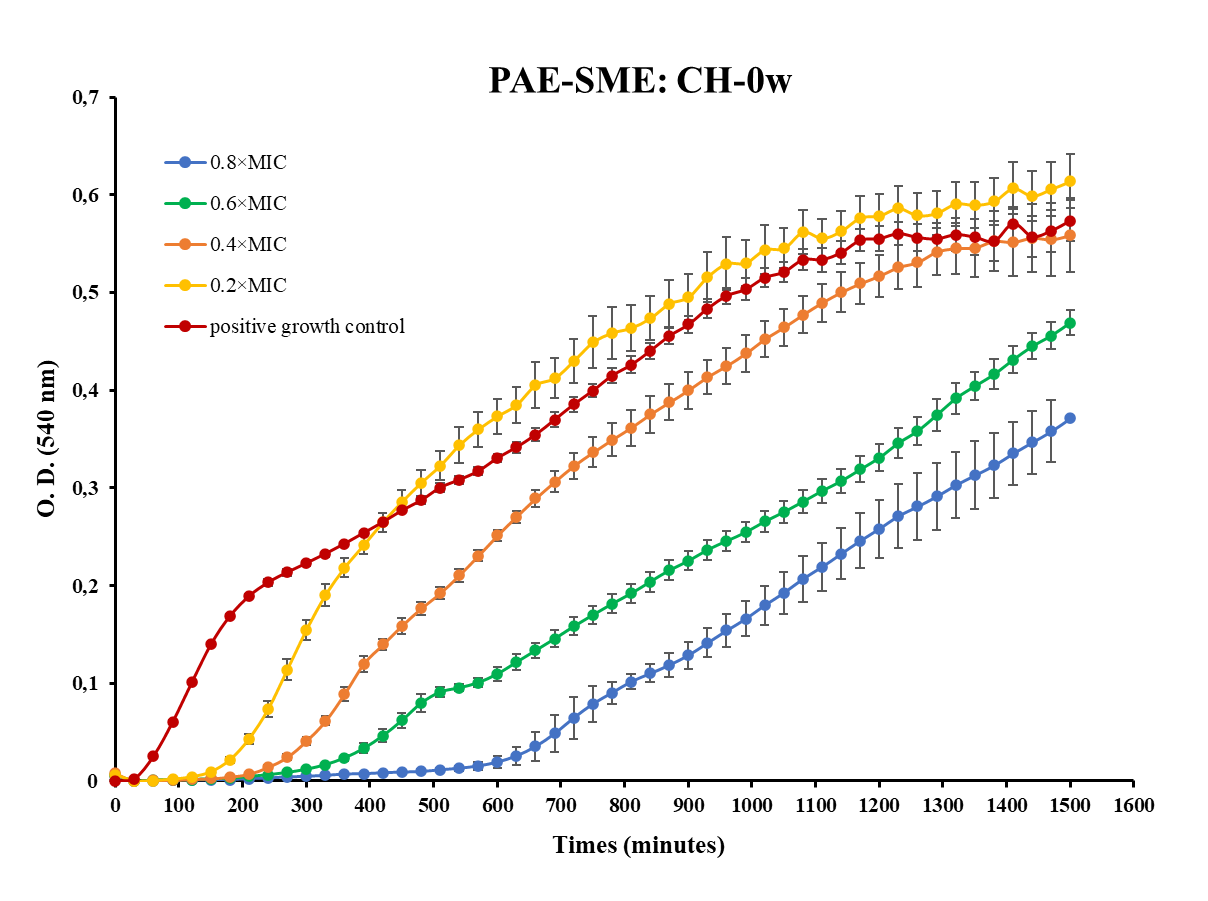

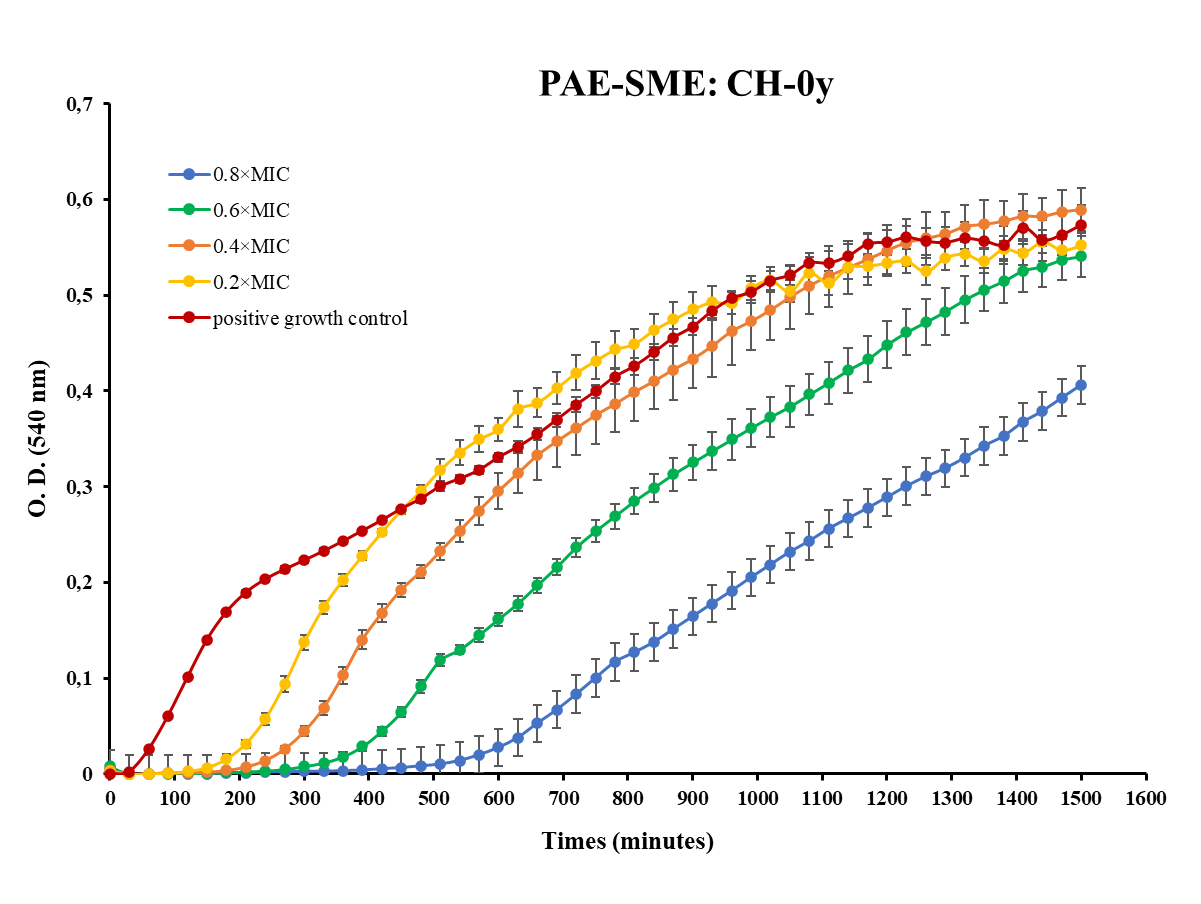


**(B)**

**(A)**


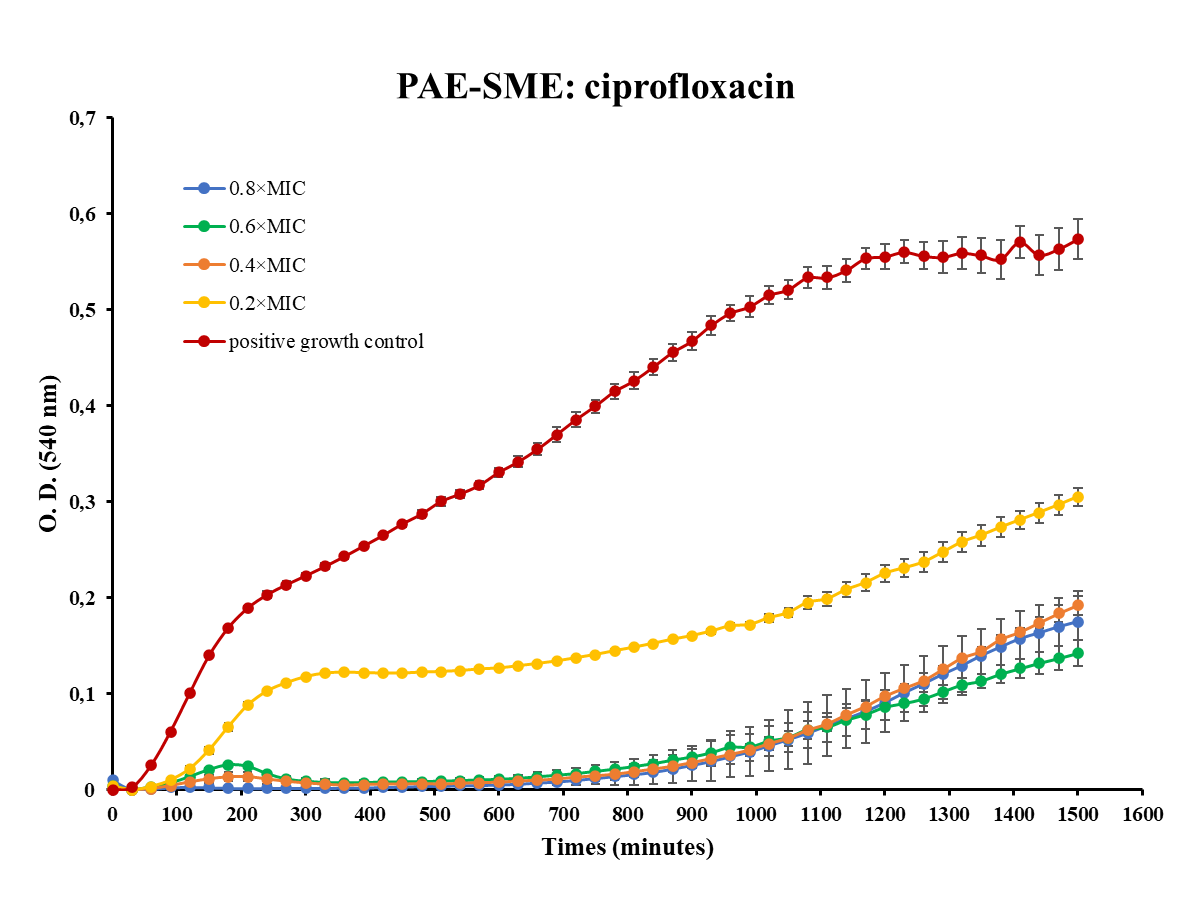

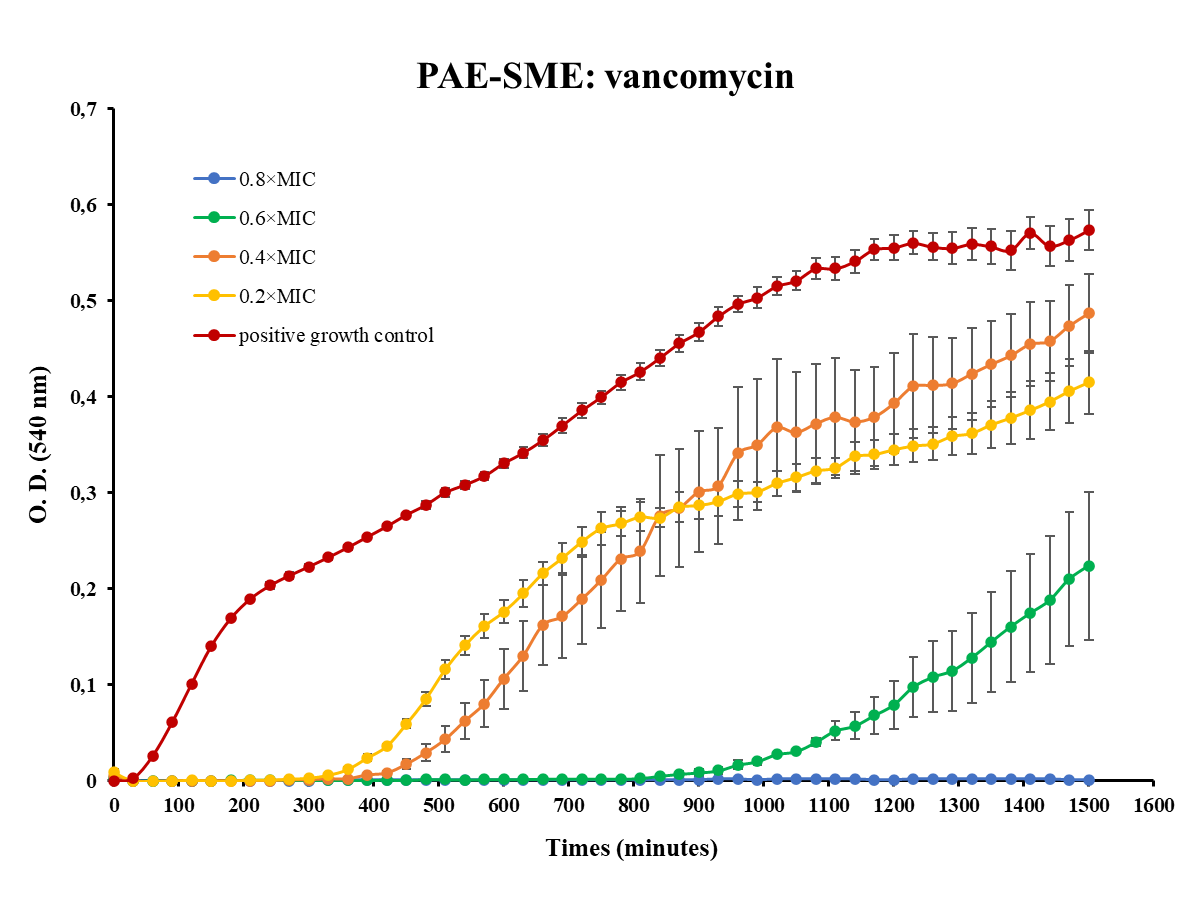


**(D)**

**(C)**

**Figure S9.** Mapping of the growth of bacterial strain, *Staphylococcus aureus* (MRSA), ATCC 43300 after 2 hours of exposure at 1×MIC of CH-0y **(A)**, CH-0w **(B)**, and conventional drugs, vancomycin **(C)**, and ciprofloxacin **(D)**. The bacterial strain was subsequently cultivated in a medium with 0.8, 0.6, 0.4, 0.2×MIC concentrations of included compounds. The growth of bacterial biomass was spectrophotometrically detected at wavelength 540 nm. The number of replicates, *n* = 3.

(a)

**
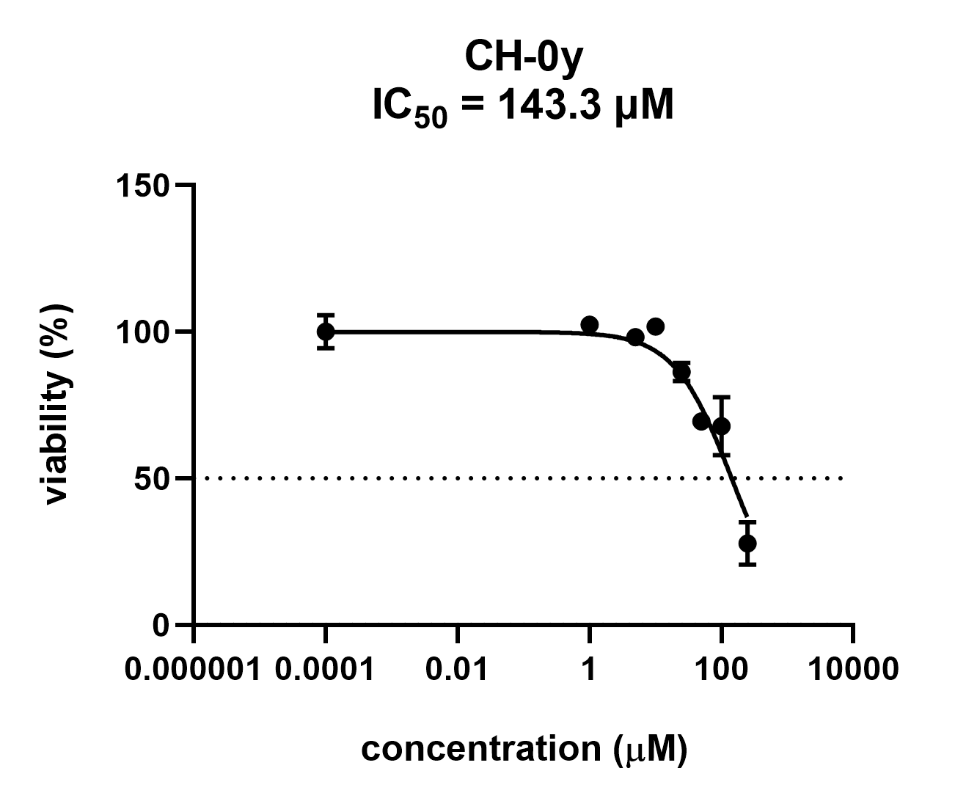

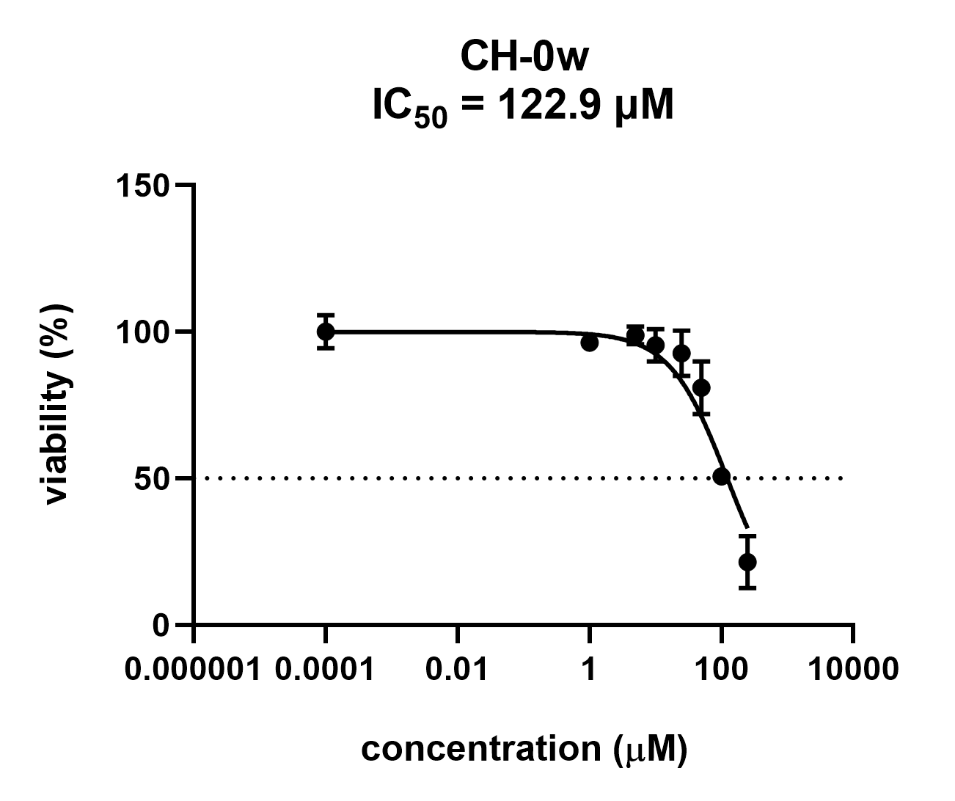
**

**(B)**

**(A)**

**Figure S10.** *In vitro* cytotoxic effect of different incubation concentrations (1 – 250 µM) of the tested compounds, CH-0y **(A)** and CH-0w **(B)**, on HK-2 cells. A standard toxicological parameter IC_50_ was calculated by nonlinear regression from a semi-logarithmic plot of incubation concentration versus percentage of absorbance relative to untreated controls using GraphPad Prism 9 software.
